# Epilation induces hair and skin pigmentation through an EDN3/EDNRB-dependent regenerative response of melanocyte stem cells

**DOI:** 10.1038/s41598-017-07683-x

**Published:** 2017-08-04

**Authors:** Huirong Li, Lilv Fan, Shanpu Zhu, Myung K. Shin, Fan Lu, Jia Qu, Ling Hou

**Affiliations:** 1 Laboratory of Developmental Cell Biology and Disease, School of Ophthalmology and Optometry and Eye Hospital, Wenzhou Medical University, Wenzhou, China; 2 State Key Laboratory and Key Laboratory of Vision Science of Ministry of Health and Zhejiang Provincial Key Laboratory of Ophthalmology, Wenzhou, 325003 China; 3 Genetically Engineered Models Department, Merck Research Laboratories, Rahway, NJ 07065 USA

## Abstract

In response to various types of injury, melanocyte stem cells (McSCs) located in the bulge of hair follicles can regenerate mature melanocytes for hair and skin pigmentation. How McSCs respond to injury, however, remains largely unknown. Here we show that after epilation of mice, McSCs regenerate follicular and epidermal melanocytes, resulting in skin and hair hyperpigmentation. We further show that epilation leads to endogenous EDN3 upregulation in the dermal papilla, the secondary hair germ cells, and the epidermis. Genetic and pharmacological disruption of the EDN3 receptor EDNRB *in vivo* significantly blocks the effect of epilation on follicular and epidermal melanocyte regeneration as well as skin and hair hyperpigmentation. Taken together, these results indicate that epilation induces McSCs activation through EDN3/EDNRB signaling and in turn leads to skin and hair hyperpigmentation. The findings suggest that EDN/EDNRB signaling may serve as a potential therapeutic target to promote repigmentation in hypopigmentation disorders.

## Introduction

Mammalian melanocytes are generated from neural crest-derived precursors (melanoblasts) that migrate along characteristic pathways to various destinations including hair follicles and epidermis or dermis^1–3^. The precursors also migrate into the bulge region of developing hair follicles where they persist as self-renewing melanocyte stem cells (McSCs) and regenerate melanocytes during the physiological hair cycle^4^. Human hair follicles also contain a special type of amelanotic melanocyte precursors in the outer root sheath (ORS) that represent a reservoir of cells capable of replenishing melanocytes in the epidermis, such as during repigmentation of vitiliginous lesions for instance in response to UVB irradiation^5^ or during wound healing in the absence of other sources for melanocyte regeneration^6^. Therefore, elucidating the processes and molecular mechanisms of how follicular melanocyte precursors respond to injuries may have broad clinical significance for an effective treatment of hypopigmenting disorders such as Vitiligo, most of which show a lack of epidermal melanocytes in skin but not amelanotic melanocyte precursors in hair follicles.

Human and mouse hair follicles share the same essential features of organization and function^7^. Nevertheless, hair growth, melanocyte populations and distribution, and expression of melanogenic enzymes differ between human and mouse skin^8^. For instance, hair growth on the human scalp is strikingly asynchronous while mouse pelage hair goes through synchronized molting stages. Furthermore, human hair follicles contain different pigment cell subpopulations that include undifferentiated amelanotic pigment cells (in bulge, outer root sheath and peripheral matrix) and three kinds of melanogenically active melanocytes (in infundibulum, sebaceous gland, and hair bulb)^8^. By comparison, mouse hair follicles normally contain undifferentiated melanocytes only in the bulge region and differentiated melanocytes in the bulb^5^. Perhaps most important is the difference in melanocyte distribution. In human skin, melanocytes persist in the interfollicular epidermis. In mice, however, with the exception of some special locations, they are absent in interfollicular epidermis and associated only with hair follicles^9, 10^. As a result, human skin pigmentation is determined mostly by epidermal interfollicular melanocytes, while in mice, it is determined by follicular melanocytes^11, 12^.

Given a mouse’s frequent molting and hair regeneration, skin pigmentation in mice is coupled with the hair cycle. During the growing stage (during mid- to late anagen), the hair follicle actively generates pigment and the skin appears black^13^. During the regressing phase (catagen) and throughout the resting phase (telogen), melanogenesis is switched-off and skin pigmentation is eventually lost^14^. Consequently, the apparent pigmentation of mouse hairy skin, made visible after hairs are clipped, is directly coupled with the anagen stage of the hair cycle^15^. In fact, as McSCs in the lower permanent portion (LPP) of the hair follicle become activated and divide only during early anagen, pigmentation of the mouse hairy skin is coupled with McSC activation^5, 11, 16^.

McSCs in the bulge of mouse hair follicles are similar to undifferentiated amelanotic melanocytes in the upper hair follicle reservoir of human hair follicles^5^. In response to injury, such as excisional wounding or UVB irradiation, McSCs are capable of migrating from hair follicles to the epidermis where they differentiate into functional epidermal melanocytes^17^. Much as other forms of skin injury, epilation can induce prompt entry into anagen and lead to hair regeneration^18^. Epilation-induced hair regeneration is thought to be mediated by an autonomous mechanism in each follicle, with early apoptosis in the bulge leading to activation of hair germ progenitors^19^. Recently, several elegant studies revealed that epilation-induced hair regeneration depends on the density of hairs plucked per surface area and so responds to a form of quorum sensing^20, 21^. These studies indicate that in response to epilation, hair germ progenitors regenerate hair follicles and that McSCs restore melanocytes in the regenerating hair follicles. Nevertheless, the mechanisms leading to McSC activation after epilation remain unclear.

To gain insight into this problem, we here compared melanocyte regeneration during physiological hair cycling with that induced by epilation. Using mice, we observed that epilation not only induces McSC proliferation in hair bulges but also regeneration of epidermal melanocytes that are not usually found during physiological hair regeneration. We further show that EDN3/EDNRB signaling is activated by epilation and disruption of EDNRB signaling can block the effect of epilation on McSC proliferation, regeneration of epidermal melanocytes, and hair and skin hyperpigmentation. The results provide detailed insights into the regulation of McSCs after epilation and may become important for the design of therapeutic approaches to repigmentation after various types of injuries.

## Results

### Epilation induces hair and skin hyperpigmentation in C57BL/6J mice

**Figure 1:**
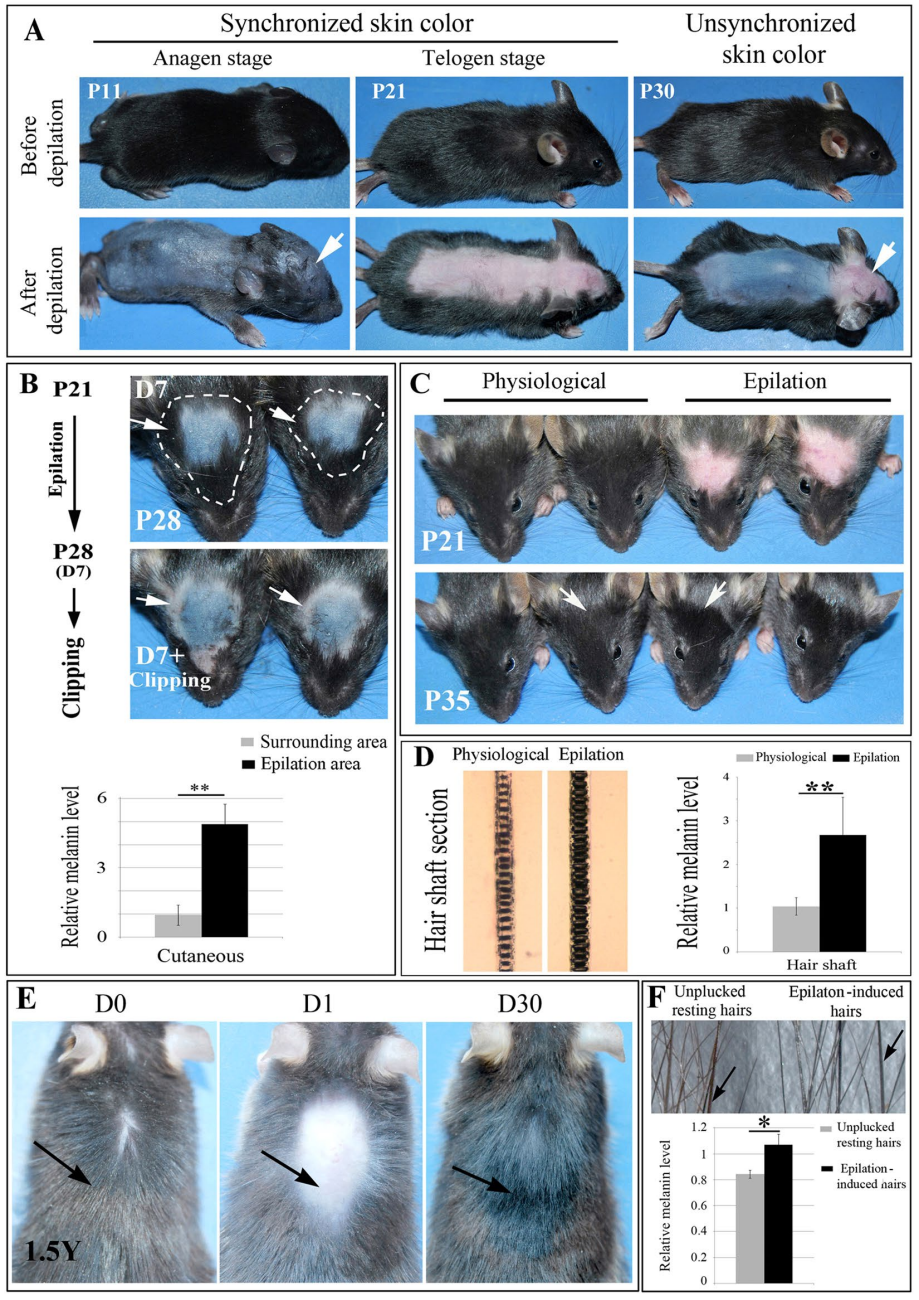
Epilation-induced skin and hair hyperpigmentation in C57BL/6 mice. (**A**) Images of control mice at the indicated ages before (upper panels) and immediately after (lower panels) hair clipping to reveal skin pigmentation. Note that during the first postnatal hair cycle, the colors of scalp and back skin differ. Arrows point to different color of scalp skin at P11 and P30. (**B**) Scalp of mice epilated at P21 and observed at P28 before (upper panels) or after clipping along the white dotted line (lower panels). Arrows point to the skin color of the clipped area. (**C**) Scalp hair pigmentation after epilation at P21. Note hyperpigmentation 14 days after epilation. Arrows indicate the different color between physiological hairs and epilation-induced regenerated hairs. (**D**) P35 scalp hair shafts and melanin levels from control mice and mice epilated at P21. (**E**) Back hairs of a 1.5 year-old mouse before and after epilation. Arrows indicate the change of hair color before and after epilation. (**F**) Back hair shafts (upper panel) and melanin levels (lower panel) from the epilated area and the surrounding area of a 1.5 year-old mouse 30 days after epilation. Arrows indicate the different color between physiological hairs and epilation-induced regenerated hairs. *Indicates *p* < 0.05, **indicates p < 0.01.

Generally, hair follicles on the dorsal skin of mice display a synchronized first hair cycle^15^. The hair follicles on the entire dorsal skin are typically in anagen from postnatal day 1–12, in catagen from day 16–19, and in telogen thereafter until the next anagen^11, 22, 23^. To visualize skin pigmentation changes during the postnatal hair cycle, we depilated the dorsal trunk and head regions of C57BL/6J mice at postnatal day 11 (P11) (anagen VI stage in first hair cycle), P21 (telogen stage), or P30, respectively. As shown in Fig. 1A, the entire skin was homogeneously black at P11 and homogeneously pink at P21. Intriguingly, however, at P30, scalp skin was still pink while back skin was black (Fig. 1A). These data indicate that although McSCs in scalp hair follicles differentiate into mature melanocytes during the first hair cycle, they are not activated to regenerate melanocytes at a time point when back hair follicles are already in anagen of the second hair cycle. We, therefore, tested whether epilation might activate McSCs in the scalp at this time point. This epilation treatment significantly induced expression of inflammatory factors, but their level is much lower than that induced by wound injury, suggesting that epilation induces only a mild form of skin injury (Fig. S1). Interestingly, when scalp hairs of C57BL/6J mice were epilated at P21 (as mentioned, in the telogen stage of the first hair cycle), scalp pigmentation was significantly increased (6.06 ± 1.5 fold, n = 9) already 7 days later, at P28 (Fig. S2A,B) and the epilation area produced a pigmented island (Fig. 1B). Correspondingly, the epilation-induced regenerating hairs were hyperpigmented (2.68 ± 0.87 fold, n = 5) compared with hairs of non-epilated controls (Fig. 1C,D). These results indicate that in the scalp, epilation can induce premature skin repigmentation and hair hyperpigmentation.

Because hairs physiologically regenerate on the back of C57BL/6J mice during the first postnatal hair cycle (Fig. 1A), we also wondered whether epilation would induce changes in hair regeneration on the back. Indeed, 7 days after epilation at P21, the epilated area was hyperpigmented compared with the unepilated one (Fig. S2C,D), as were the individual regenerated hairs (Fig. S2E). These data suggest that epilation-induced hyperpigmentation is not restricted to the scalp. Given these observations, we also asked whether the effects of epilation-induced hyperpigmentation would still work in old mice. Intriguingly, the regenerating hairs in the back of 1.5 year-old mice became significantly hyperpigmented 30 days after epilation (1.07 ± 0.08 fold, n = 3), compared with the unepilated, resting hairs (0.84 ± 0.03) (Fig. 1E,F). In addition, HPLC analysis showed that epilation induces eumelanin synthesis (1.79 ± 0.09 fold, n = 3) instead of pheomelanin synthesis (0.97 ± 0.12 fold, n = 3) in back hairs (Fig. S3). This suggests that epilation induces activation of McSCs to generate either more mature melanocytes, or it increases melanin synthesis in mature melanocytes in hair bulbs of aged mice.

### Epilation-injury stimulates McSC proliferation and induces generation of dermal and epidermal melanocytes and expression of melanogenesis-related genes

**Figure 2:**
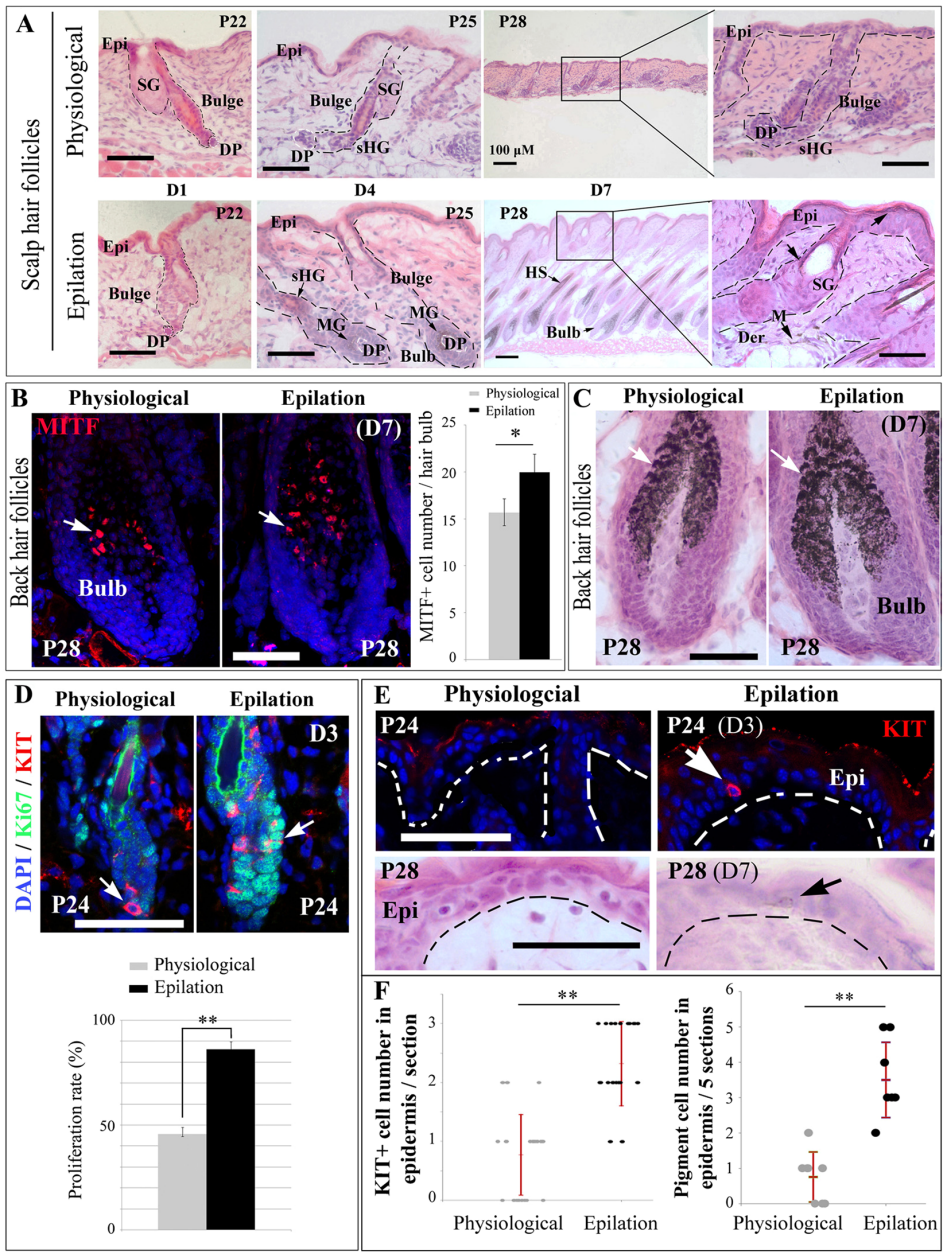
Epilation stimulates McSC proliferation, leads to regeneration of epidermal melanocytes and induces expression of melanogenesis-related genes. (**A**) H&E staining of distinct stages of scalp hair cycling 1, 4, and 7 days after epilation. C57BL/6 mice were epilated at P21 and the histology of hair follicles was analyzed at the indicated days. (**B**,**C**) Anti-MITF immunostaining (**B**) and H&E staining (**C**) of back hair follicles from control mice at P28 and age-matched mice epilated at P21. Arrows indicate the MITF+ cells (**B**) and melanin granules in the hair bulb (**C**). The 3D images of anti-MITF immunostaining are shown in Fig. S10. (**D**) Immunostaining of anti-KIT and Ki67 in back hair follicles of C57BL/6J mice 3 days after epilation. Note that epilation induces proliferation of McSCs. Arrows indicate the KIT+ cells in the hair bulge. (**E**) Anti-KIT immunostaining of back skin from control mice at P24 and age-matched mice epilated at P21 (upper panels) and H&E staining of back skin (lower panels) on day 7 after epilation. Note KIT-positive cells in the epidermis 3 days after epilation and pigmented melanocytes in the epidermis 7 days after epilation. Arrows indicate the KIT+ cell (upper panels) and pigmented cell (lower panels). (**F**) graphs show the number of Kit+ (left panel) and pigmented cells (right panel) in the epidermis 3 days and 7 days after epilation, respectively. DP, dermal papilla; Epi, epidermis; Der, dermis; M, melanocyte; MG, melanin granules; SG, sebaceous gland; sHG, secondary hair germ. Bar, 50 μm. *Indicates *p* < 0.05, **indicates *p* < 0.01.

Given that melanogenesis is coupled to anagen^24^, we examined whether epilation-induced pigmentation in the scalp is caused by anagen induction. As shown in Fig. 2A, the epilation-induced hair follicles started anagen immediately, while in non-epilated control mice, the hair follicles remained at telogen for a longer time period. Hence, the quiescent hair follicle McSCs responded to epilation by becoming activated. Seven days after epilation, pigmented melanocytes were observed not only in mature hair bulbs but also in the dermis, orifices of hair follicles, and the interfollicular epidermis (Fig. 2A). Correspondingly, melanocyte-specific markers such as PMEL17 and TYR were detected in the epilation-induced pigmented regions but not in control hair follicles (Fig. S4A). In addition, western blot data showed that the levels of the melanogenesis-related proteins TYRP1 (4.22 ± 0.96 fold, n = 6) and TYR (9.75 ± 2.76 fold, n = 6) were both increased significantly by epilation (Fig. S4B and S9). The results suggest that epilation not only activates McSCs but also induces regeneration of melanocytes to populate interfollicular areas.

In the back skin, 7 days after epilation at P21, the total melanocyte number in each hair bulb (20 ± 1.9) was significantly increased compared with physiological regeneration (15.7 ± 1.4) (Fig. 2B). Correspondingly, there were also more melanosomes in the epilation-induced regenerated hair bulbs than in physiologically regenerated hair bulbs (Fig. 2C and Fig. S4C,D). These data suggest that epilation induced McSC hyperproliferation during the first postnatal hair cycle. Indeed, 3 days after epilation, the McSCs’ proliferation rate was higher (86.2 ± 3.5% of cells were Ki67-positive after epilation compared to 45.6 ± 3.4% of cells positive for Ki67 in controls) (Fig. 2D). Interestingly, KIT-positive cells were not found in the epidermis but they existed in the hair bulge 1 day after epilation and clearly present in the epidermis 3 days after epilation (Fig. 2E and Fig. S5). The number of Kit+ cells in the epilated epidermis (2.32 ± 0.72/section, n = 4) is increased significantly compared with physiological epidermis (0.77 ± 0.68/section, n = 4) (Fig. 2F). Correspondingly, 7 days after epilation, pigmented cells were also found in the epidermis (Fig. 2E,F). Furthermore, quantitative RT-PCR 7 days after epilation revealed induction of many melanogenesis-related genes, including *Tyr* (2.84 ± 1.13 fold over control, n = 3), *Tyrp1* (3.89 ± 0.72 fold, n = 3), *Mitf* (3.47 ± 0.38 fold, n = 3), *Sox10* (2.13 ± 1.13 fold, n = 3), *Pax3* (3.18 ± 0.43 fold, n = 3) and *Ednrb* (3.28 ± 0.62 fold, n = 3) (Fig. S6A). Taken together, these results suggest that epilation induces McSCs proliferation, migration into the epidermis, and stimulation of the melanogenesis program.

### Epilation induces EDN3 expression in hair follicles and epidermis

**Figure 3:**
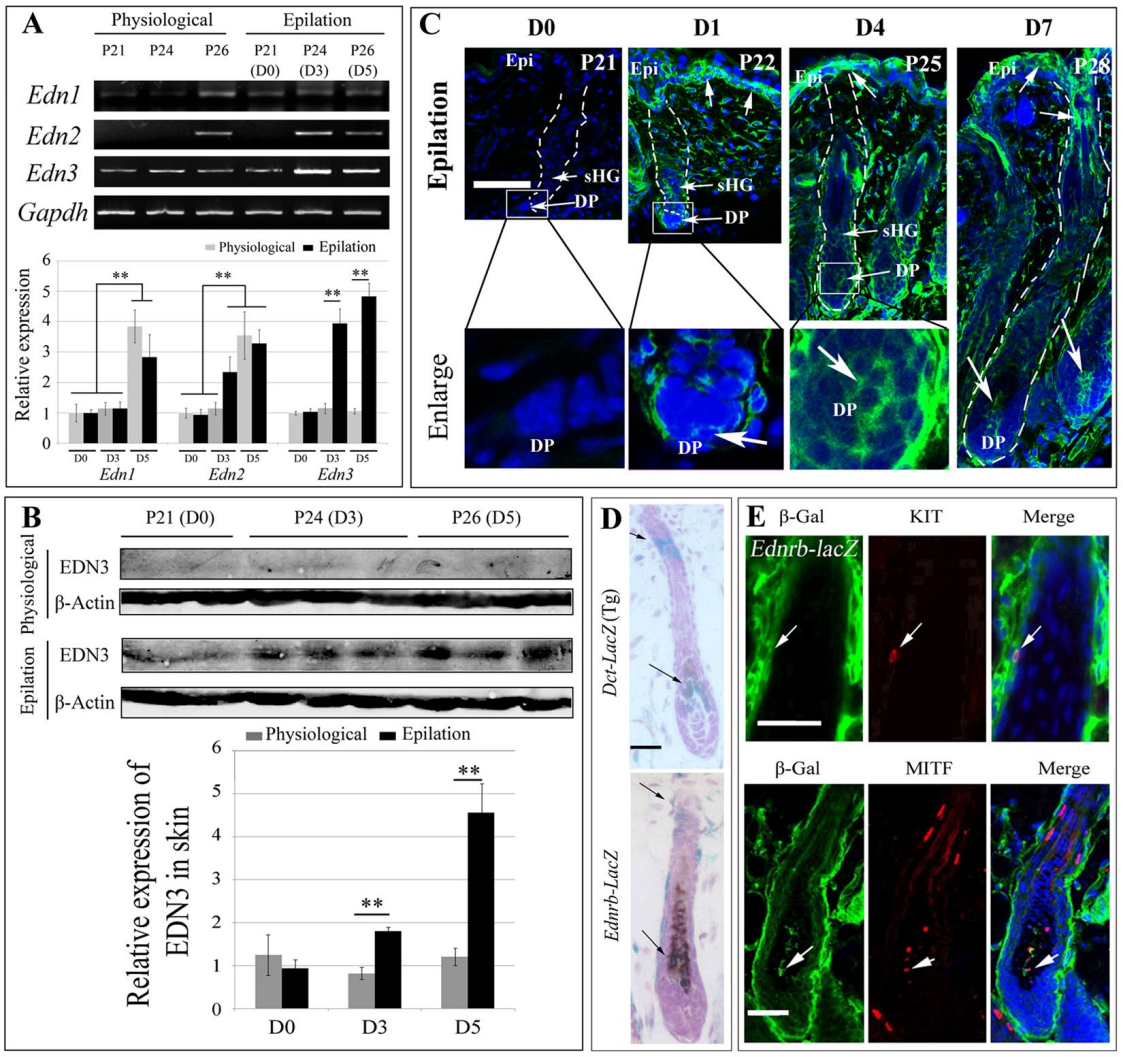
Epilation induces expression of endothelin-3 (Edn3) in C57BL/6 J mice. (**A**) RT-PCR and qRT-PCR analysis for *Edns* expression changes in skin after epilation. Mice were epilated at P21 and gene expression was analyzed at the indicated days. Full-length gels are shown in Fig. S11. (**B**) Western blotting analysis for protein expression of EDN3 in the skin. Full-length gels are shown in Fig. S12. (**C**) Immunofluorescence of EDN3 on scalp skin at 0, 1, 4, and 7 days after epilation. Arrows indicate EDN3+ cells in the hair follicles. (**D**) X-gal staining of hair follicles from *Dct*-lacZ transgenic mice (upper panels) and *Ednrb* ^*lacZ*/+^ mice (lower panels). Arrows indicate the lacZ+ cells in the hair bulges and bulbs. (**E**) Immunohistochemistry of *β-*Gal, KIT, and MITF in pigmented hair follicles from postnatal day 7 *Ednrb* ^*lacZ*/+^ mice. Arrows indicate KIT and *β-*Gal co-labeled cells in the bulge (upper panels) and MITF and *β-*Gal co-labeled cells in the hair bulb (lower panels). Epi, epidermis; DP, dermal papilla; sHG, secondary hair germ. Bar: 50 μm. **Indicates p < 0.01.

Among the genes induced by epilation, EDNRB was of particular interest because of its well known involvement in the regulation of the melanocyte lineage^1, 12^. Recently, the expression of its ligands *Edn1* and *Edn2* has been reported to be increased during physiological hair anagen, but its ligand *Edn3* expression was not affected^12^. Therefore, we wondered whether epilation of back hairs would induce these ligands. In confirmation of the above results, we found that during physiological regeneration, the expression of *Edn1* and *Edn2* were increased in the back skin at P26, but *Edn3* expression showed no change (Fig. 3A and Fig. S11). Surprisingly, however, epilation not only induced the expression of *Edn1* and *Edn2* but also that of *Edn3* (Fig. 3A), whose encoded product, EDN3, has previously been shown to be involved in melanocyte development^1, 25, 26^. Hence, we focused our study on EDN3 and its signaling pathway. We found that the level of EDN3 protein was significantly increased in epilated skin (D0, 0.94 ± 0.2 fold; D3, 1.81 ± 0.09 fold; D5, 4.57 ± 0.67 fold; n = 6) but remained low in control skin (D0, 1.25 ± 0.47 fold; D3, 0.83 ± 0.14 fold; D5, 1.21 ± 0.2 fold; n = 6) (Fig. 3B and Fig. S12). Immunohistochemistry showed that on epilation day 1, EDN3 protein was increased in the dermal papilla, epidermis, and secondary hair germ. On day 7, it was also increased in the orifices of hair follicles (Fig. 3C). The regions with high epilation-induced EDN3 levels were in close contact with the McSCs in the secondary hair germ (sHG) or with hair bulb melanocytes. Using adult heterozygous *Ednrb* ^*lacZ*^^/+^ mice, we then found that EDNRB is expressed in pigmented melanocytes of hair bulbs and in sHG, similar to the expression of *Dct*-lacZ in hair follicles (Fig. 3D). Furthermore, immunostaining data showed that *β-*Gal positive cells were co-labeled with KIT-positive cells in sHG and with MITF-positive cells in hair bulb (Fig. 3E), suggesting that EDNRB is expressed in McSCs and melanocytes. As expected, 7 days after epilation, western blot analysis and immunostaining data showed that *Ednrb*-lacZ expression is significantly increased in melanocytes (Figs S6B,C and S13). These results suggest that epilation induces EDN3, which then stimulates its receptor EDNRB to regulate McSCs and melanocytes.

EDN3 is well known for its effects on stimulating the growth and differentiation of melanocyte precursors^1, 25, 26^. Recently, transgenic overexpression of EDN3 has been reported to prevent hair graying caused by repeatedly epilation^27^, suggesting that EDN3 also affects McSCs. To investigate the effect of EDN3 on McSCs, we isolated DCT+ cells from E16.5 wildtype epidermis and stimulated them with EDN3 or BQ788 (an EDNRB inhibitor). As shown in Fig. S7A, stimulation with EDN3 significantly increases the proliferation rate of the DCT+ cells (from 43.7 ± 2% up to 61.4 ± 7.5%, n = 5), but this effect of EDN3 was totally blocked by BQ788 (26.4 ± 1.56%, n = 5). Based on the facts that for unpigmented cells, DCT is a specific marker of McSCs and that there are no mature melanocytes in the E16.5 epidermis^4, 28^, the result suggests that EDN3/EDNRB is required for McSC proliferation.

### *Ednrb* is required for epilation-induced hair and skin pigmentation

**Figure 4:**
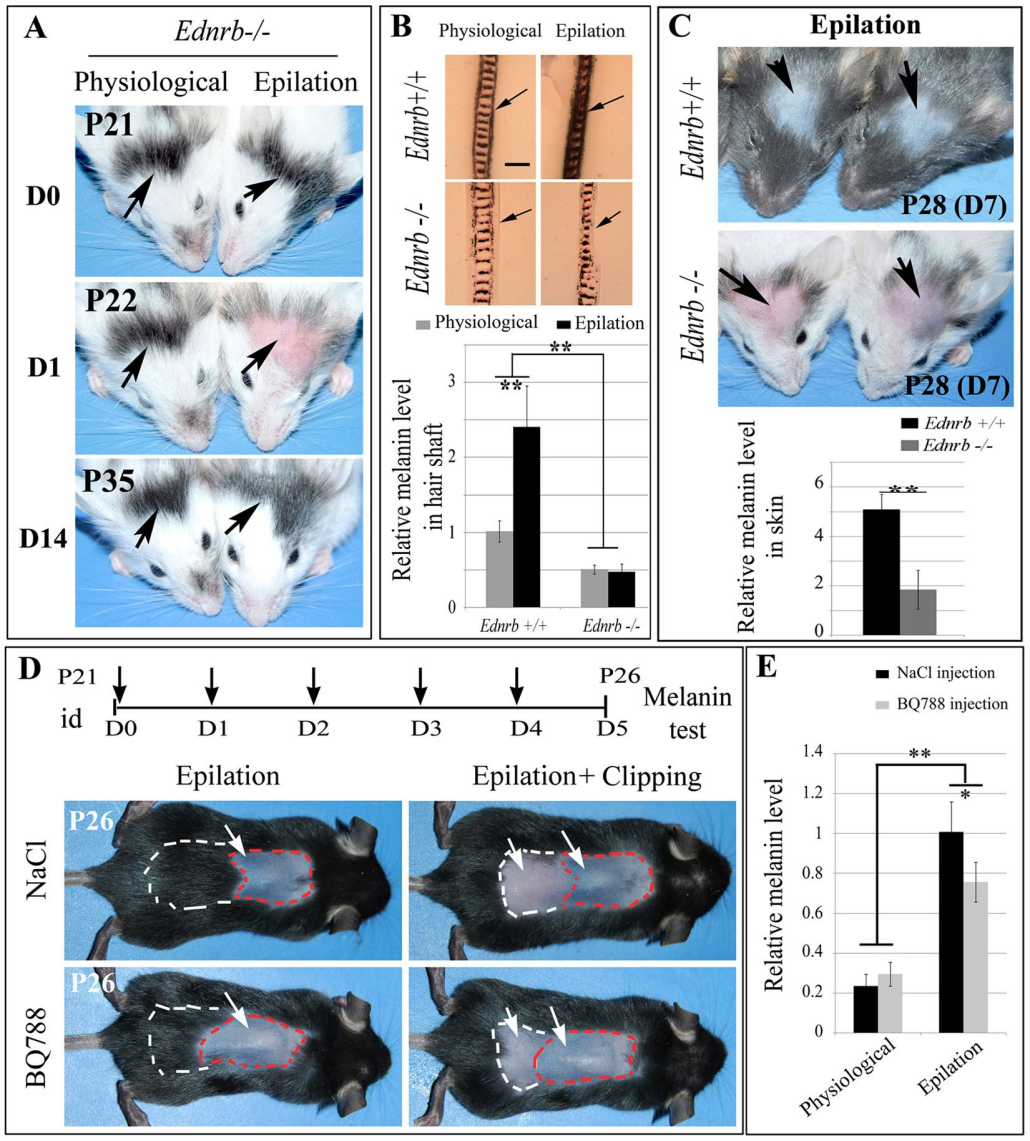
Genetic and pharmacological disruption of *Ednrb* blocks epilation-induced hair and skin hyperpigmentation. (**A**) Hair pigmentation of *Ednrb*−/− mice before and after epilation at P21. Arrows point to the epilated (right panels) and unepilated (left panels) pigmented hairs of *Ednrb*−/− scalp. (**B**) Histological section of hairs (upper panels) and melanin content (lower panel) of the hair shafts of wildtype and *Ednrb*−/− mice before and after epilation. Arrows indicate the decrease of melanin in the hair shaft of *Ednrb*−/− mice. (**C**) Pigmentation of P28 scalp of wildtype and *Ednrb*−/− mice after epilation at P21. Arrows point to the epilated area of wildtype (upper panels) and *Ednrb*−/− mice (lower panels). (**D**, **E**) Back skin of P26 mice epilated at P21 and intracutaneously injected with either physiological saline (upper panels) or BQ788 (lower panels) on the days indicated by the vertical arrows (**D**). The white arrows point to the skin color of epilated and clipped areas. (**E**) The graph shows relative melanin levels of the back skin. Note that on day 5 after epilation along the red dotted line, clipping along the white dotted line shows the surrounding hairs as controls for physiological regeneration of hair follicular pigmentation. id, intradermal injection. *indicates *p* < 0.05; **indicates *p* < 0.01.

To evaluate the importance of EDNRB signaling for epilation-induced skin hyperpigmentation, we used homozygous *Ednrb* ^*lacZ/lacZ*^ (*Ednrb*−/−) mice. Such mice are largely free of pigment cells but retain pigmented spots at the base of the head and the tail. Fourteen days after epilation in the head region, regenerating hairs were hyperpigmented in wildtype mice (2.41 ± 0.55 fold, n = 5) but not in *Ednrb*−/− mice (Fig. 4A,B). In unepilated *Ednrb*−/− mice, the melanin levels of hair shafts were lower (0.51 ± 0.06 fold, n = 5) compared with those in control wildtype mice (1.02 ± 0.14 fold, n = 5) (Fig. 4B). In addition, skin repigmentation was significantly increased in wildtype mice, but only slightly in *Ednrb*−/− mice (Fig. 4C). The skin melanin levels induced by epilation were significantly lower in *Ednrb*−/− mice (1.85 ± 0.79 fold, n = 9) compared with those in wildtype mice (5.1 ± 0.62 fold, n = 9). These results suggest that deletion of *Ednrb* disrupts epilation-induced hair hyperpigmentation and skin repigmentation. Furthermore, loss of EDNRB is associated with hair graying. The melanin levels of pigmented hair shafts of *Ednrb*−/− mice were reduced between one month of age (0.96 ± 0.1 fold, n = 4) and up to 12 months of age (0.37 ± 0.12 fold, n = 4), in contrast to their levels in hair shafts of wildtype mice (1.96 ± 0.17 fold, n = 4 at one month; 2.15 ± 0.43 fold, n = 4, at 12 months) (Fig. S7B).

To examine whether EDNRB signaling is required for epilation-induced hyperpigmentation in the back skin, we performed intradermal injection of BQ788, after epilation of wildtype mice. As shown in Fig. 4D, while control intradermal injection of NaCl after epilation allowed for skin hyperpigmentation (from 0.23 ± 0.06 fold up to 1.0 ± 0.15 fold, n = 3), injection of BQ788 significantly decreased the epilation-induced skin pigmentation (0.76 ± 0.1 fold, n = 3). This finding suggests that pharmacological disruption of EDNRB can block epilation-induced skin hyperpigmentation in the back skin. Taken together, these data suggest that EDNRB signaling is required for epilation-induced hyperpigmentation.

### EDNRB affects McSC proliferation in hair follicles and regulates melanogenesis-related gene expression

**Figure 5:**
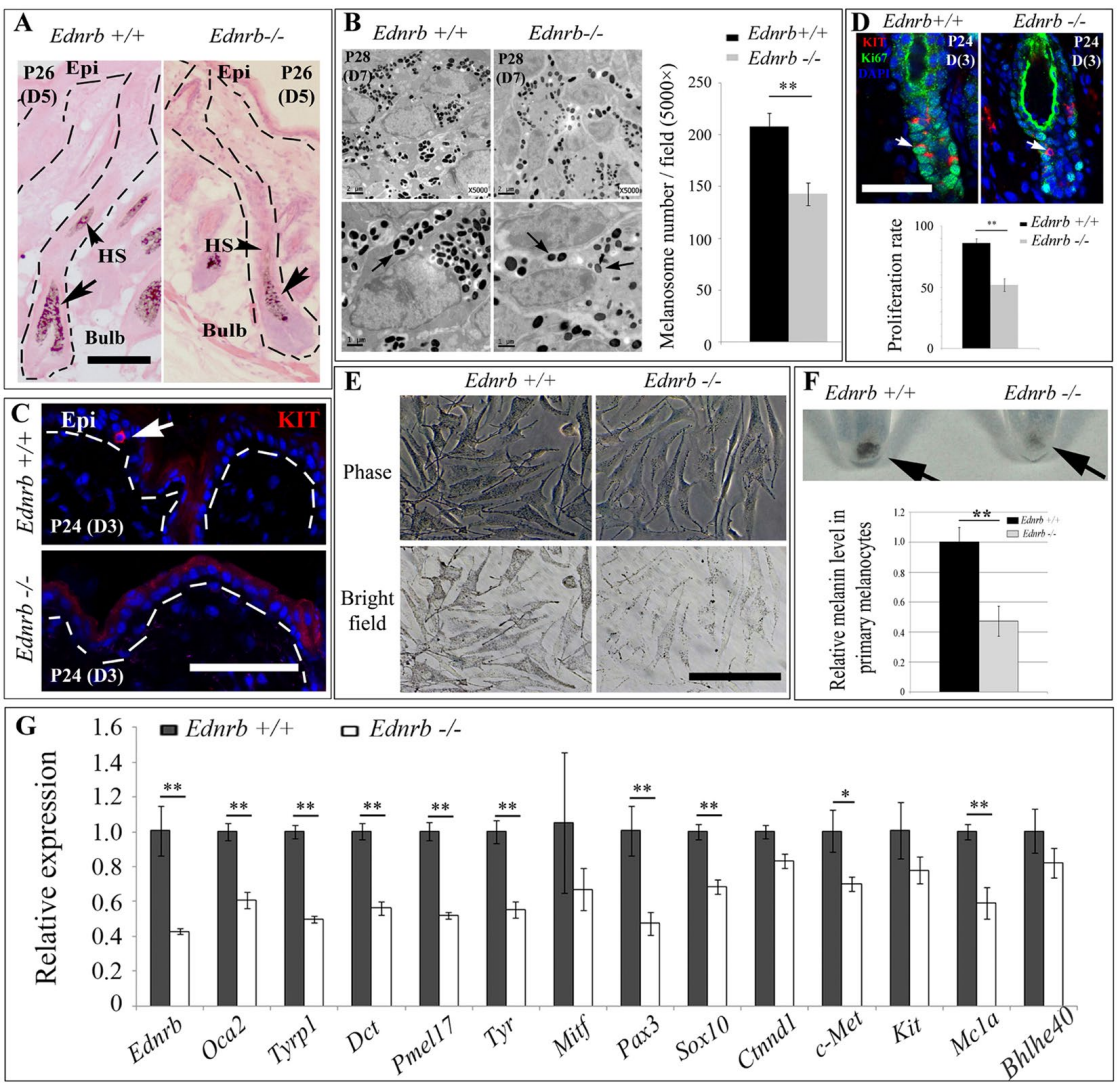
Loss of EDNRB blocks McSC migration into epidermis, reduces melanocyte proliferation in hair bulb and decreases expression of some melanogenesis-related genes. (**A**) Histology of scalp hair follicles from wildtype and *Ednrb*−/− mice 5 days post-epilation. Arrows indicate melanin granules in the hair bulb and pigmented hair bulb. (**B**) Melanosomes of wildtype and *Ednrb*−/− mice in the hair bulbs 7 days post-epilation revealed by transmission electron microscopy (TEM). Arrows indicate the melanosome. (**C**) Anti-KIT immunostaining of scalp from wildtype and *Ednrb*−/− mice 3 days after epilation. Note lack of KIT-positive cells in *Ednrb*−/− epidermis 3 days after epilation. (**D**) Anti-KIT and Ki67 immunostaining of hair follicles from wildtype and *Ednrb*−/− mice 3 days post-epilation (upper panels) and the quantitative graph of melanocyte proliferation in the hair bulb (lower panel). (**E**) Images of primary cultures of melanocytes from wildtype and *Ednrb*−/− mice and (**F**) corresponding cell pellets (upper panel) and melanin content (lower panel). Arrows point to the cell pellet. (**G**) qRT-PCR analysis for expression of melanogenesis-related genes in scalp skin of wildtype and *Ednrb*−/− mice 7 days after epilation. Epi, epidermis; HS, hair shaft. Bar, 50 μm. *Indicates p < 0.05; **indicates p < 0.01.

To examine how the lack of *Ednrb* decreases skin pigmentation, we histologically analyzed the pigmentation of hair follicles on the scalp (which, as mentioned, remains pigmented in *Ednrb*−/− mice in contrast to the back). As shown in Fig. 5A, on day 5 after epilation, hair bulbs and hair shafts of *Ednrb*−/− mice were normally present in the scalp but were hypopigmented compared with those of wildtype mice, suggesting that loss of EDNRB does not affect the hair cycle but decreases melanocyte numbers or melanin synthesis in the bulb. Indeed, 7 days after epilation, the melanosome number of *Ednrb*−/− mice was much lower than that of wildtype mice (Fig. 5B). In addition, melanocyte-specific markers such as PMEL17 and TYR could not be detected in the dermis, orifices of hair follicles, or the interfollicular epidermis of *Ednrb*−/− mice 5 days after epilation, even though they were present at low levels in hair bulbs (Fig. S7C). Furthermore, the McSC marker KIT and pigmented melanocytes remained undetectable even 7 days after epilation (Fig. 5C and Fig. S7D). These results suggested that EDNRB is required for epilation-induced epidermal melanocyte regeneration.

To analyze how the loss of EDNRB decreases the melanin level of hair bulbs, we then quantified melanocyte numbers in each hair bulb of wildtype and *Ednrb*−/− mice 7 days after epilation. The number of MITF-positive cells upon epilation was lower in each hair follicle of *Ednrb*−/− mice (11.7 ± 2.2) compared to hair follicles in wildtype mice (15.7 ± 1.43) (Fig. S7D). This result suggested that loss of EDNRB could block epilation-induced proliferation of melanocyte lineage cells. Recently, Takeo *et al*. found that during physiological hair regeneration, EDNRB signaling is also required for McSCs proliferation and maintenance^29^. Nevertheless, whether EDNRB affects epilation-induced McSC proliferation was still unknown. Since EDN3 is expressed in the dermal papilla in close proximity to mature melanocytes in the hair bulb, we tested whether EDN3/EDNRB signaling affects McSC proliferation in the bulge or stimulates melanin synthesis in follicular melanocytes. In the scalp, anti-KIT and Ki67 immunostaining data showed that 3 days after epilation, McSC proliferation in each hair bulge of *Ednrb*−/− mice (52 ± 5.2%) was lower than in corresponding bulges of wildtype mice (86.2 ± 3.5%) (Fig. 5D). This suggests that EDNRB is also required for epilation-induced McSC proliferation in the hair bulge.

To gain further insights into the underlying mechanisms, we isolated melanocytes for *in vitro* culturing. As shown in Fig. 5E, the pigmentation of *Ednrb*−/− primary melanocytes from pigmented head hair follicles of P6 *Ednrb*−/− mice was lower (0.47 ± 0.1 fold, n = 5) than that of wildtype primary melanocytes (1.0 ± 0.1 fold, n = 5). Similar results were obtained in cell pellets or when melanin content was directly measured (Fig. 5F). We then analyzed whether EDNRB signaling would affect the expression of melanogenesis-related genes. As shown in Fig. 5G, on day 7 after epilation, the expression of melanogenesis-related genes was decreased in *Ednrb*−/− scalp, including expression of *Pax3* (0.47 ± 0.07 fold, n = 3), *Tyr* (0.55 ± 0.05 fold, n = 3) and *Tyrp1* (0.5 ± 0.02 fold, n = 3). This suggests that EDN3/EDNRB signaling is involved in both melanocyte proliferation and melanogenesis during epilation-induced regenerative responses of McSCs. Taken together, these findings indicate that epilation induces melanogenesis-related gene expression through EDN3/EDNRB signaling and in turn leads to skin and hair hyperpigmentation.

## Discussion

Epilation can induce prompt entry into anagen and lead to hair regeneration^18^. Classical studies showed that epilation leads to hair keratinocyte apoptosis, inflammatory changes, and finally hair regeneration^19, 21^. It was unclear, however, whether physiological hair regeneration and epilation-induced hair regeneration would follow the same mechanisms. In fact, our results revealed distinct differences. First, during the initial postnatal hair cycle, epilation-induced regenerating hairs were hyperpigmented compared with physiologically regenerated hairs. Moreover, while under physiological conditions, hair graying gradually develops as mice age, epilation induced repigmentation in the new hairs rather than more graying hairs. Second, epilation induced the presence of KIT-positive cells in the epidermis. These cells might well represent McSCs as KIT has been reported to be expressed in McSCs^30^ and loss of EDNRB, thought to specifically affect the melanocyte lineage in the skin^29^, led to the disappearance of these cells in the epidermis. Recently, it has also been shown that in *Edn1* transgenic mice, epidermal melanocytes are generated after hair epilation but not without epilation^29^. These observations all suggest that epilation-injury prompts McSC migration into the epidermis. Third, epilation induces upregulation of melanogenesis-related genes in melanocytes and also induces *Edn3* whose expression is not induced during physiological hair regeneration^12^. Collectively, these observations indicate that epilation not only induces prompt entry of hair follicles into anagen but also activates McSC to generate epidermal melanocytes and induces melanogenesis by mechanisms that differ from those encountered during normal hair cycling. Interestingly, McSCs can also respond to wound healing or UVB irradiation by migrating from the hair follicle niche through the upper follicular epithelium to the basal layer of the epidermis^17^. This feature of McSCs is similar to that of undifferentiated amelanotic melanocytes in the upper hair follicle reservoir of human hair follicles, which represent a reservoir that can replenish melanocytes in the epidermis when responding to UVB irradiation or wound healing^5^.

**Figure 6:**
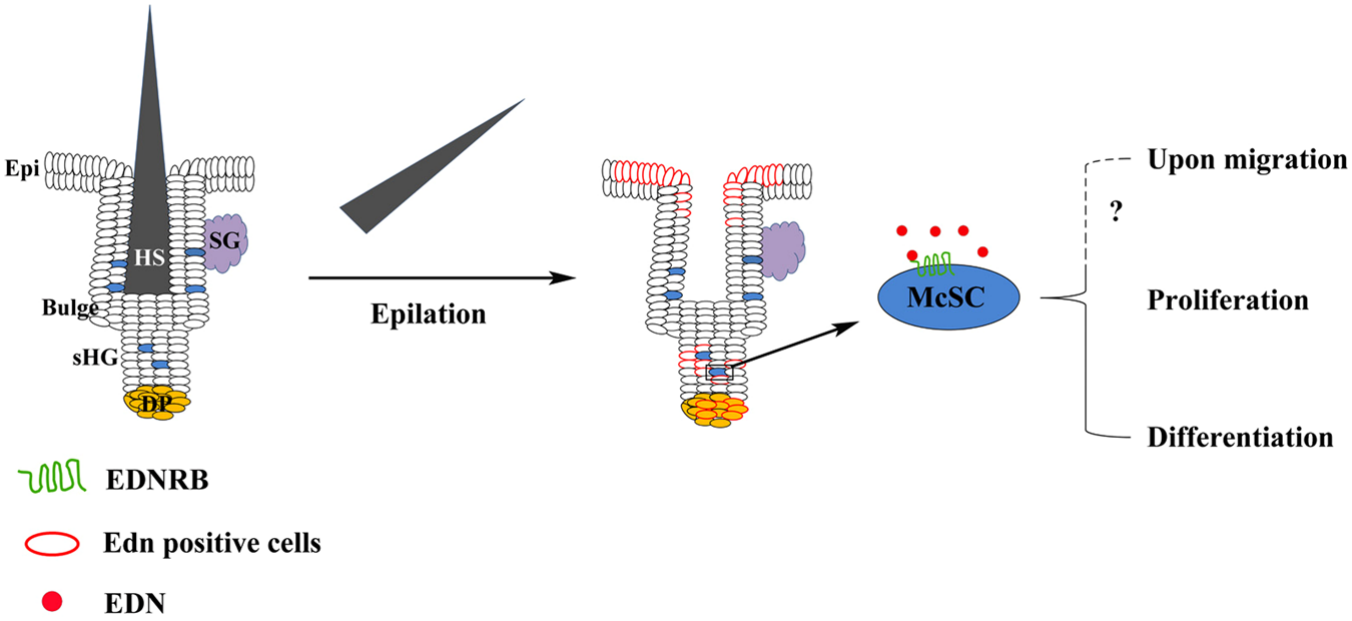
Schematic illustration of the role of EDN/EDNRB signaling in epilation-induced skin and hair pigmentation. After epilation, expression of EDNs is increased in epidermis, secondary hair germ and dermal papilla. McSCs respond to the increased EDNs secreted from surrounding cells, resulting in activation of the EDNRB signaling pathway. As a result, McSCs proliferate, likely migrate along the hair follicles into epidermis and differentiate into mature melanocytes for melanogenesis. Epi, epidermis; DP, dermal papilla; HS, hair shaft; sHG, secondary hair germ.

In hair follicles, the activity of McSCs is regulated by both intrinsic factors, such as the transcription factors MITF and SOX10^30^, and extrinsic factors, including WNT, KITL, and TGFβ^31, 32^. It has been shown that WNT activation in McSCs drives their differentiation into pigment-producing melanocytes and that EDNRB signaling mediates melanocyte expansion induced by β-catenin stabilization in epithelial stem cells^12^. It is well known that EDNRB signaling plays very important roles in melanocyte development during embryogenesis, such as in melanoblast migration and differentiation^1, 25, 33–35^. Recently, evidence from transgenic and conditional knockout animal models indicated that EDN/EDNRB signaling is involved in McSC differentiation^29, 36^. Consistently, pigmented scalp hairs of *Ednrb*−/− mice are hypopigmented compared to those of wildtype mice and become dramatically gray during the first year of life, suggesting that EDNRB signaling is involved in McSC maintenance and melanogenesis in adult mice. However, whether injury, or which type of injury, might induce activation of EDNRB signaling in hair follicles was still unclear. Here we found that epilation-injury induces EDN3 expression in the secondary hair germ, dermal papilla, and epidermis, and activation of EDN3/EDNRB signaling in hair follicles. These regions are in close contact with McSCs and melanocytes, suggesting that EDN3/EDNRB signaling might regulate McSCs activity. Indeed, genetic and pharmacological disruption of *Ednrb* blocks epilation-induced regeneration of epidermal melanocytes, decreases melanocyte proliferation in hair bulbs and reduces skin pigmentation and hair hyperpigmentation. In contrast, transgenic overexpression of EDN3 can prevent hair graying caused by repeated epilation^27^. This role of EDN3/EDNRB signaling can also be observed in epidermal pigmentation following skin wounding. Overexpression of EDN1 in postnatal mouse skin induces migration of McSCs to the epidermis^29^. Selective ablation of EDN1 in murine epidermis, however, does not alter melanocyte homeostasis in newborn skin^37^, probably because EDN2 and/or EDN3 compensate for the lack of EDN1. Taken together, our findings suggest that the activity of McSCs induced by injury is at least in part mediated by EDN3/EDNRB signaling as schematically shown in Fig. 6, although it is currently unknown how epilation upregulates Edn3 expression.

On the dorsal skin of mice, hair follicles initially grow in a nearly synchronized manner. Our results show that the synchronization of hair follicle growth over the entire dorsal skin is not maintained for the second hair cycle. Rather, at P30, the dorsal trunk skin is effectively repigmented, while the dorsal head skin forms a depigmented island although the corresponding hair shafts, and hence the head coats, remain pigmented. That hair follicles of the back and scalp differ during the second hair cycle has also been reported in *Bcl2*−/− mice, in which McSCs are not normally maintained. In contrast to the results presented here for normal mice, *Bcl2*−/− mice have hypopigmented hair shafts in the back but normally pigmented hair shafts in the scalp^38^, either because the trophic conditions on the back and scalp are different, or because the earlier entry into the second hair cycle in the trunk predisposes the corresponding McSCs to a more rapid loss. In the future, it will be important to determine how McSC activities are differentially regulated in scalp and back skin during regeneration in adult mice.

In conclusion, we hope that the presented results showing that epilation can induce premature McSC activation will prompt future studies of the role of injury for therapeutic repigmentation in disorders of localized depigmentation.

## Materials and Methods

### Animals

All animal experiments were carried out in accordance with the approved guidelines of the Wenzhou Medical University Institutional Animal Care and Use Committee. C57BL/6J mice were obtained from The Jackson Laboratory*. Ednrb* ^*lacZ/lacZ*^ mice carrying a P1 transgene capable of rescuing their megacolon (though not their pigmentary defect) have been described^39^ and were kept on a C57BL/6 background. They are here referred to as *Ednrb*−/− mice for simplicity. *Dct*-lacZ transgenic mice were kindly provided by Dr. William Pavan (NIH). The experimental protocol was approved by the Wenzhou Medical University Animal Care and Use Committee (Permit Number: WZMCOPT-090316).

### Epilation-induction and HE staining

Hairs of postnatal day 21 (P21) wildtype (WT) and *Ednrb*−/− mice were removed using an epilatory paste under ketamine and xylazine anesthesia. For excisional wounds, the dorsal fur of P21-old mice was clipped. After mice were anaesthetized with ketamine and xylazine, 1 cm^2^ area of skin was excised. After 3 days, the wounding area was subjected to analyze gene expression. For histological analysis, the skins were fixed in 4% paraformaldehyde (PFA) overnight at RT and paraffin-embedded sections (5 μm thickness) were prepared according to standard procedures. The stages of hair cycling were determined according to a comprehensive guide^11^.

### Transmission electron Microscopy

Pigmented skins were fixed in 2.5% glutaraldehyde for 3 hours at RT, then post-fixed in 1% osmium tetroxide for 1 hour at 37 °C. After washing with PBS, the skins were treated with 1% phosphotungstic acid and 1% sodium uranyl acetate for 1 hour at 37 °C, dehydrated through acetone series and epoxy resin-acetone mixture at 37 °C, and then embedded in epoxy resin at 45 °C. Semi-thin sections were cut and stained with methylene blue (Sigma) to localize the melanin granules in the hair bulbs. Ultrathin sections were cut and mounted on grids, and the specimens were examined and photographed with a H-7500 transmission electron microscope (Hitachi).

### Intradermal injection of BQ788 *in vivo*

BQ788 (Sigma) was dissolved in physiological saline to a concentration of 1 mg/ml. Mice were injected intradermally at 5 mg/kg body weight everyday for 5 days after hair epilation. Injection was performed in the central area of the epilated region.

### X-gal staining and immunofluorescence

The process of X-gal staining has been described^35^. For immunofluorescence, 14-µm cryosections were prepared and stained according to standard procedures using the following antibodies as indicated: mouse anti-HMB45 (1: 200, Abcam), mouse anti-TYRP1 (1: 100, Abcam), goat anti-TYR (1: 50, Santa Cruz Biotech), mouse anti-β-galactosidase (1: 100, Promega), rat anti-KIT (1:50, B&D), rabbit anti-Ki67(1: 100, B&D), mouse anti-Ki67 (1: 100, B&D) and rabbit anti-EDN3 (1: 100, Abcam). Appropriate Alexa 488- or 594-conjugated secondary antibodies (Invitrogen) were used at RT for 1 hour. The sections were examined and photographed with a Zeiss fluorescence microscope. For analysis of melanocyte numbers in hair bulbs, 40 µm cryosections were prepared and incubated with rabbit anti-MITF (1: 200, gift from Dr. Arnheiter) primary antibody at 4 °C for 24 hours. Alexa 594-conjugated secondary antibodies (Invitrogen) were used at RT for 2 hours. The sections were examined and photographed with a two-photon Zeiss fluorescence microscope. For quantification of *Ednrb-*lacZ expression in hair bulb melanocytes, we used Image J software to measure the fluorescence signal. Significance was analyzed using a paired Student’s-t test.

### RT-PCR and Western Blot

RNA from head skins was extracted using 0.5 ml of Trizol reagent (Invitrogen) according to the manufacturer’s protocol. RT-PCR was performed according to standard protocols using a reverse transcriptase kit and random primers (Promega). PCR products were size-fractionated by 2% agarose gel electrophoresis. Realtime PCR was performed in triplicate with Power SYBR Green PCR Master Mix on a 7500 Real-Time PCR Detection System (Applied Biosystems). Relative mRNA expression levels were normalized to those of GAPDH and analyzed using the 2-DDCt method. All gene-specific primers were designed by Primer 5 Software. Primer sequences are depicted in Supplementary Table 1.

Dissected head skins were placed in RAPI protein lysis solution (Byotime, China) and lysed on ice for 10 minutes with a Micro Tissue Grinder. Samples were separated by 10% SDS-PAGE and western blots were prepared by standard procedures using either goat anti-TYR (1: 500, Santa Cruz Biotech), mouse anti-β-galactosidase (1: 800, Promega), mouse anti-TYRP1 (1: 500, Abcam) or rabbit anti-EDN3 (1: 1000, Abcam) as primary antibodies and appropriate secondary IRDye® 700CW or IRDye® 800CW-conjugated antibodies. Protein bands were scanned using a LI-COR machine. Mouse anti-α-Tubulin or anti-β-Actin antibodies (1: 2000, Santa Cruz Biotech) were used as internal controls. Protein bands were quantified using Image J software.

### Primary melanocyte and McSC cultures

Pigmented skins of P6 wildtype and *Ednrb*−/− mice were incubated with 0.25% trypsin (Biotime, China) and 1 mg/ml dispase (Invitrogen, Carlsbad, CA) in DMEM for 3 hours at 37 °C. Subcutis and outer-most layers of the skin were removed. The remainder of the skin tissue was incubated with 0.05% trypsin and EDTA for 30 min at 37 °C. Single cell suspensions were cultured for 12 days in 90% DMEM/F12 supplemented with 10% FBS; 100 nM TPA; 12 ng CT; Glutamine; Gentamicin. This yielded nearly 100% pure pigmented melanocytes as judged by MITF staining. Melanocytes were then kept in 90% DMEM/F12 medium +10% FBS for 24 hours and exposed to 20 nM EDN3 for 8 days before use.

For isolating E16.5 wildtype epidermal DCT+ cells, the cultures were prepared as described in Nishikawa-Torikai *et al*. (2011)^40^. In brief, dorsal skin from E16.5 embryos from wildtype mice was incubated in PBS containing 5 mM EDTA for 1 hour at 37 °C, and the dermis was removed using a pair of fine forceps under a microscope. The epidermis was further treated with DMEM (Gibco) containing 10% fetal calf serum, 2 mg/ml collagenase P (Roche), and 2 mg/ml dispase (Invitrogen) for 15 minutes at 37 °C and then dissociated by pipetting to obtain a single-cell suspension. The suspension was centrifuged at 1000 rpm to obtain single cells. The cells were cultured in RPMI 1640 medium (Sigma-Aldrich) containing 10% fetal bovine serum (FBS), 1 mg/ml insulin, 1 mM phosphoethanolamine, 1 mM ethanolamine, 50 ng/ml SCF, 2.5 ng/ml FGF2, 100 nM αMSH, 100 nM TPA. After 5 days, these growth factors and TPA were exchanged for 20 nM EDN3 or 20 nM EDN3 combined with 5 nM BQ788. BQ788 was replenished every other day. For analysis of McSC proliferation, cells were fixed with 4% PFA for 25 minutes at room temperature, and rabbit anti-DCT antibody (1: 100, Bioworld) and mouse anti-Ki67 antibody (1: 100, Millipore) were used to label McSCs and proliferating cells, respectively.

### Melanin content analysis

Melanin levels in cultured cells were measured as described previously^41^. In similar ways, melanin levels were determined from dissected head skins digested with 0.25% trypsin at 37 °C for 1 hour, lysed with RAPI on ice for 10 minutes using a Micro Tissue Grinder, centrifuged at 10000 × g for 10 min, and solubilized in 200 μl of 1N NaOH and 10% dimethyl sulfoxide for 2 h at 80 °C. Hair shafts were dissected using microscissors and melanin prepared in similar ways. Solubilized melanin was assessed by absorbance at 405 nm.

For eumelanin and pheomelanin tests, HPLC analysis was used and performed as described in Ito *et al*. (2011)^42^. Briefly, hair samples were homogenized in water (10 mg/ml) with a Ten-Brocke glass homogenizer, and the resulting suspensions were then sonicated with an ultrasonic cell disrupter. 100 μl of water suspensions of samples (1.0 mg hair) was placed in 1.5 ml EP tubes, to which 375 μl 1 mol/l K_2_CO_3_ and 25 μl 30% H_2_O_2_ (final concentration: 1.5%) were added. The tubes were mixed vigorously at 25 ± 1 °C for 20 h on a test-tube mixer. The residual H_2_O_2_ was decomposed by adding 50 μl 10% Na2SO3 and the mixture was then slowly acidified with 140 μl of 6 mol/l HCl. Each reaction mixture was centrifuged at 4000 g for 1 minute, and an aliquot (20 μl) of each supernatant was directly injected into the HPLC system. H_2_O_2_ oxidation products were analyzed with an HPLC system consisting of an Agilent 1200 liquid chromatograph (Agilent, USA), an Agilent C18 column (4.6 × 250 mm, 5 μm particle size; Agilent, USA), and an Agilent UV detector at 269 nm. The mobile phase was 0.1 mol/l potassium phosphate buffer (pH 2.1)/methanol, 99:1 (v/v). Analyses were performed at 45 °C at a flow rate of 0.7 ml/min. We injected 20 ng of PTCA (Santa Cruz Biotechnology) as a standard for eumelanin. Hair samples of agouti mice were used to identify the pheomelanin peak. The relative level of eumelanin or pheomelanin was calculated by the ratio of the areas underneath the representative peaks.

### Statistical Analysis

Data are from at least three replicates for each experiment and are represented as mean ± standard error of the mean (SEM). Except for the analysis of *Ednrb* ^*lacZ*^ expression where a paired *t-*test was used, Student’s *t*-test was used to determine the significance of the differences between the population means. *P* < 0.05 was considered to be statistically significant. Significant differences between groups are indicated by *or **.

## Electronic supplementary material

Supplementary information
