# Supplementary material for: Epilation induces hair and skin pigmentation through an EDN3/EDNRB-dependent regenerative response of melanocyte stem cells
Source: Sci Rep. 2017 Aug 4;7:7272. doi: 10.1038/s41598-017-07683-x (PMC5544680; doi:10.1038/s41598-017-07683-x)
Supplement: Supplementary file 1 — Supplementary information [file 41598_2017_7683_MOESM1_ESM.doc]

**Epilation induces hair and skin pigmentation through an EDN3/EDNRB-dependent regenerative response of melanocyte stem cells**

**Huirong Li ^1,2^, Lilv Fan^1^, Shanpu Zhu^1^, Myung K. Shin^3^, Fan Lu^2^, Jia Qu^2^,, Ling Hou ^1,2^***

**Supplemental information**

**Supplemental table 1.**

**PCR primer sequences**

Tnfα-Forward CCAAATGGCCTCCCTCTCA

Tnfα-Reverse TTGCTACGACGTGGGCTACAG

Kif4- Forward CGCCATCAGAACCAGGACAAT

Kif4- Reverse TTCTTCCTCTAGCATAGAAGGATCT

C4b-Forwar CAGTGAGAAGTTGGGCCAGT

C4b- Reverse TTCAAACTGCTCAAAGCGGC

Tbx2-Forward             GGACGACCCCAAGGTGACGC

Tbx2-Reverse              CATCCGCCTCCCGGACTTGG

Oca2-Forward              ATGCGCCTAGAGAACAAAGAC

Oca2-Reverse               TAGCAGGTTTGACGGTCAGC

Pax3-Forward              TCGGCCTTGCGTCATTTCTC

Pax3-Reverse               GATGGCACCAGGTCGGATG

Mitf-Forward                CCTTCCAGCGTTTCTATGTC

Mitf-Reverse                 ACTTCTTTCAGTTTGGGTTA

Dct-Forward                  GTCCTCCACTCTTTTACAGACG

Dct-Reverse                   ATTCGGTTGTGACCAATGGGT

Tyr-Forward                  CTCTGGGCTTAGCAGTAGGC

Tyr-Reverse                   GCAAGCTGTGGTAGTCGTCT

Tyrp1-Forward             ATGAAATCTTACAACGTCCTCCC

Tyrp1-Reverse              GCACACTCTCGTGGAAACTGA

Gapdh-Forward            AGGTCGGTGTGAACGGATTTG

Gapdh -Reverse            TGTAGACCATGTAGTTGAGGTCA

SOX10-Forward AGGTTGCTGAACGAAAGTGAC

SOX10-Reverse CCGAGGTTGGTACTTGTAGTCC

Ctnnb1-Forward CGCTTGGCTGAACCATCAC

Ctnnb1-Reverse GTTCCGCGTCATCCTGATAGT

Bhlhe40-Forward CGGAGACCTGTCAGGGATGGAT

Bhlhe40-Reverse CAGCCGGTGCGGCAGTTTGTA

Mc1R-Forward TGCTTTCCGCAGCCAGGAGC

Mc1R-Reverse TCTGCCCAGCGCCCTCTGAT

Ednrb-Forward GTGGCTTCTTGGGGGTATGG

Ednrb-Reverse TCTTAGTGGGTGGCGTCATTA

c-Met-Forward CCTCTGCCGCTGCTTCAAT

c-Met-Reverse GCTGCGTAGGGTATTCGTGG

Ccl4-Forward AGCACCAATGGGCTCTGAC

Ccl4-Reverse CCATCTCCATGGGAGACACG

Edn1-Forward ACCGTATGGACTGGGAGGTT

Edn1-Reverse GGTGAGCGCACTGACATCTA

Edn2-Forward TGAACACTGCGGGACAGACA

Edn2-Reverse TGTGCTGAACCCATCAGACG

Edn3-Frward GCGCTCTGAAAGTTCGTGAC

Edn3-everse ACAGCTTTCGAGTCCTGTCG

Ccl2-Forward GCAGCAGGTGTCCCAAAGAA

Ccl2-Reverse TTGGTTCCGATCCAGGTTTTT

Ccl3-Forward TCCCAGCCAGGTGTCATTTTCC

Ccl3- Reverse GTCACCAAACAGTGTGACCAAC

**Supplemental figures**

**
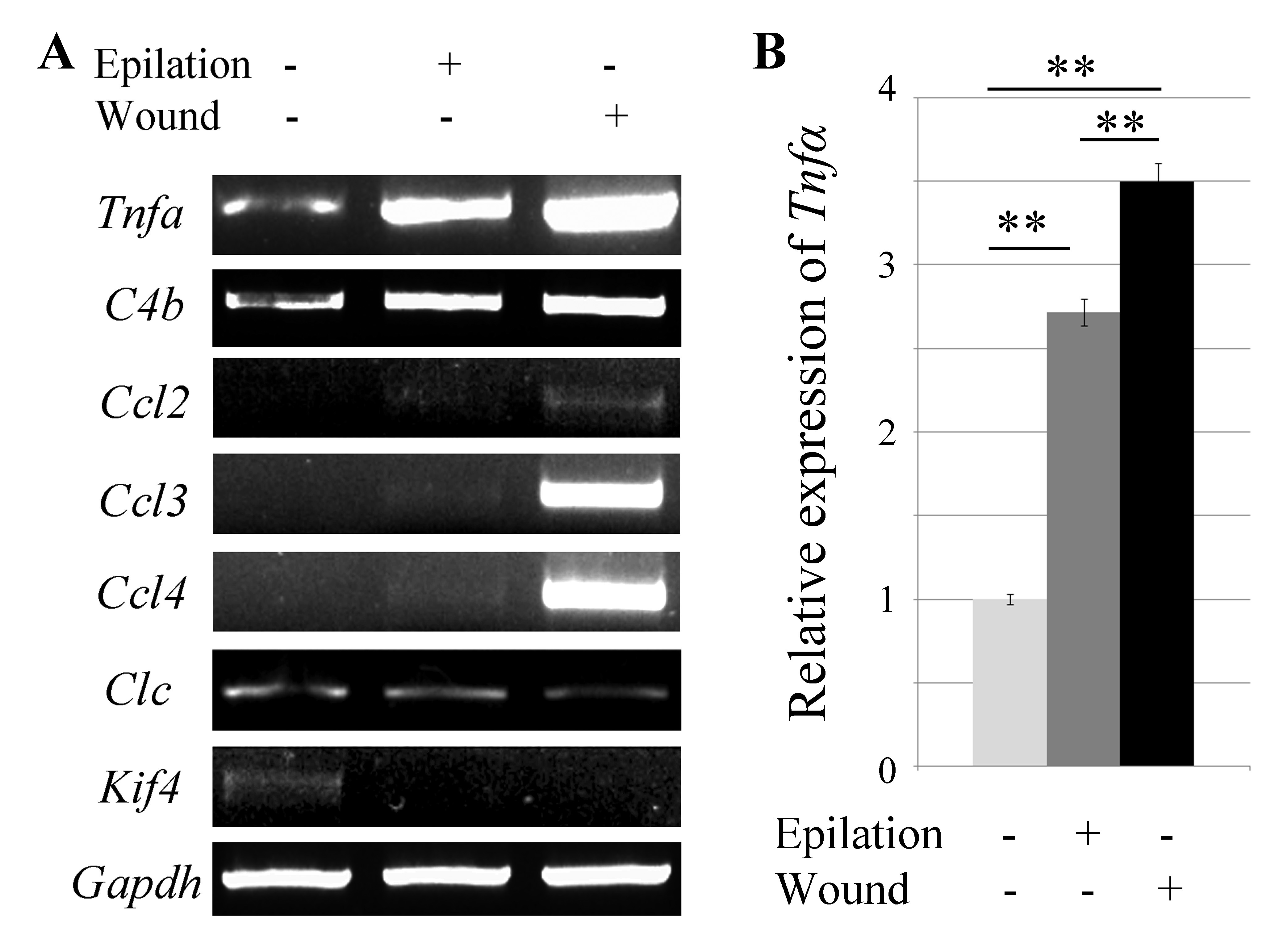
**

**Figure S1. Epilation induces expression of inflammatory-related genes.** (A) RT-PCR analysis shows the expression change of inflammatory-related genes in the back skin 3 days after epilation or wounding. Full-length gels are shown in Figure S8. (B) Quantitative real-time PCR (qRT-PCR) analysis shows the expression change of *Tnfα* in the back skin under the indicated conditions. ** indicates *p*<0.01.

**
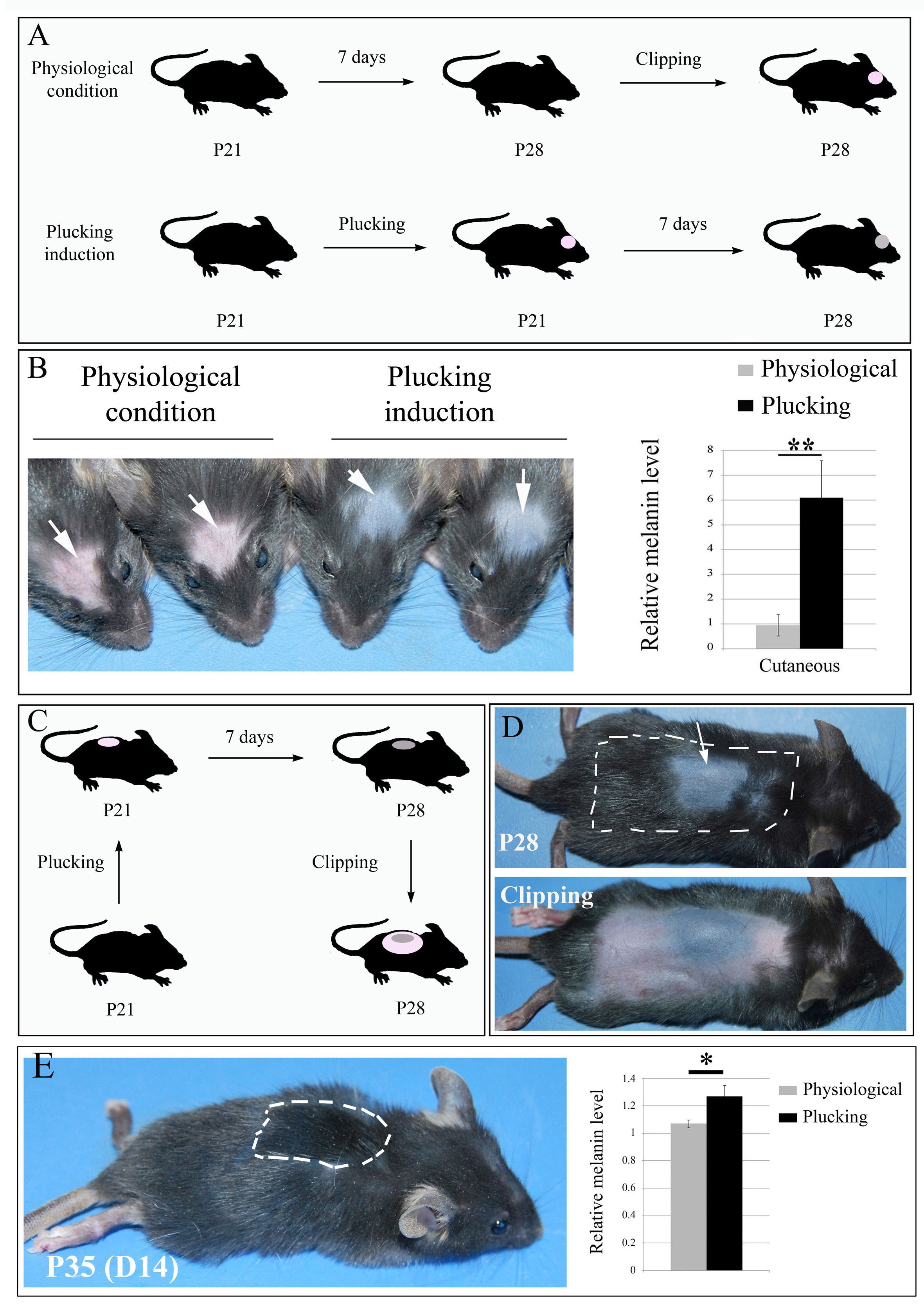
 Figure S2.** **Epilation induces scalp and back skin hyperpigmentation.** (A) The cartoon indicates the experimental epilation process in the scalp. (B) Scalp pigmentation and melanin levels 7 days after epilation at P21 or left unepilated. (C) The cartoon indicates the experimental time line for epilation and clipping on the back. (D) Back skin pigmentation 7 days after epilation at P21. (E) Back hair pigmentation 14 days after epilation at P21. * or ** indicates *p*<0.05 or *p*<0.01.

.


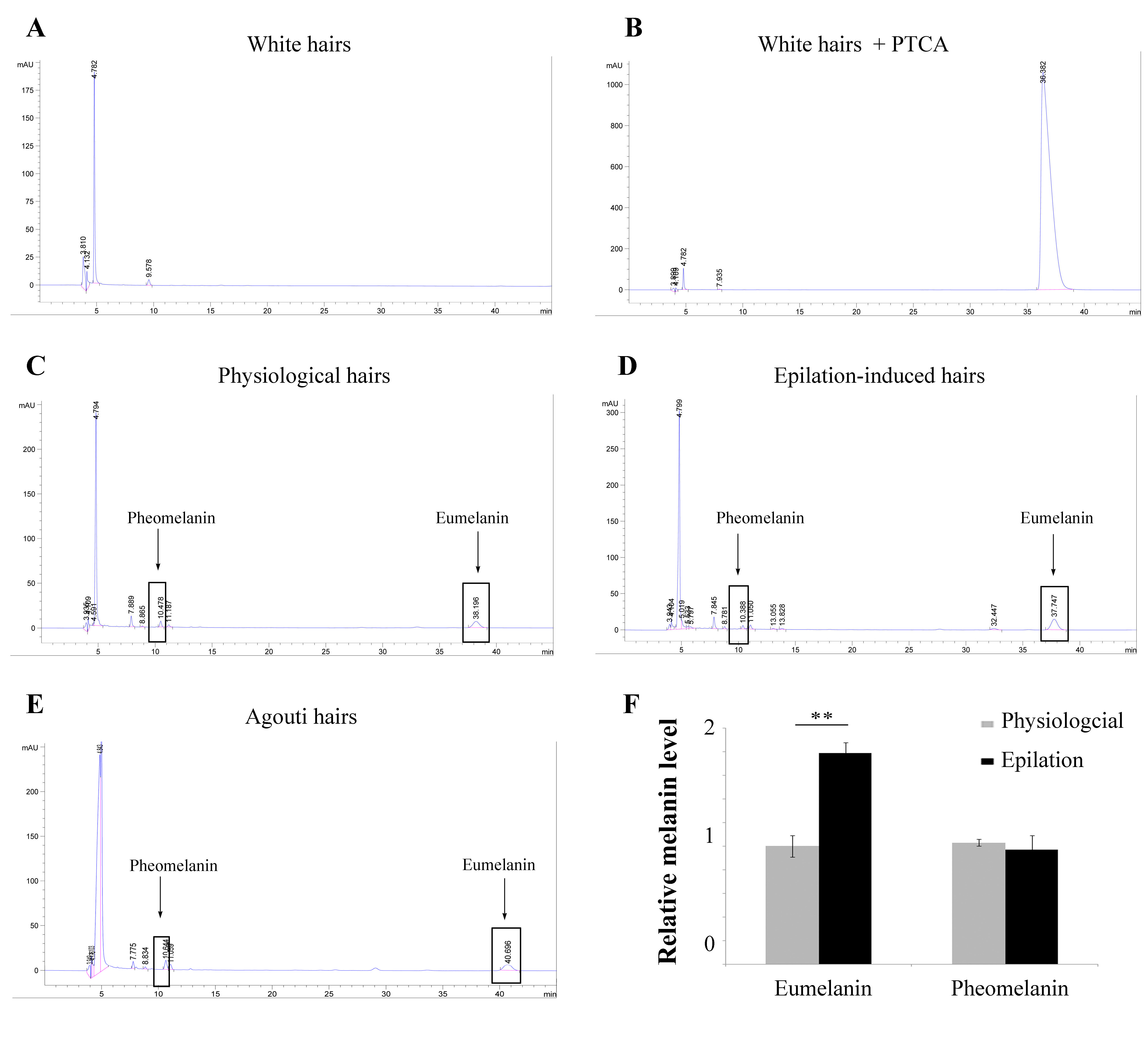


**Figure S3. Epilation leads to an increase in eumelanin production in hairs of 1.5 year-old wildtype mice.** (A-E) HPLC chromatograms of alkaline H_2_O_2_ degradation products. (A) White hairs from P21 *Ednrb-/-* mice, (B) White hairs from P21 *Ednrb-/-* mice with 20 ng PTCA reagent. (C, D) Physiological hairs (C) of 1.5 year-old wildtype mice, (D) epilation-induced hairs of 1.5 year-old mice 30 days after epilation, (E) Yellow hairs from P21 agouti mice. (F) Bar graphs show the relative levels of eumelanin or phenomelanin in the physiological hairs and epilation-induced hairs. ** indicates *p*<0.01.


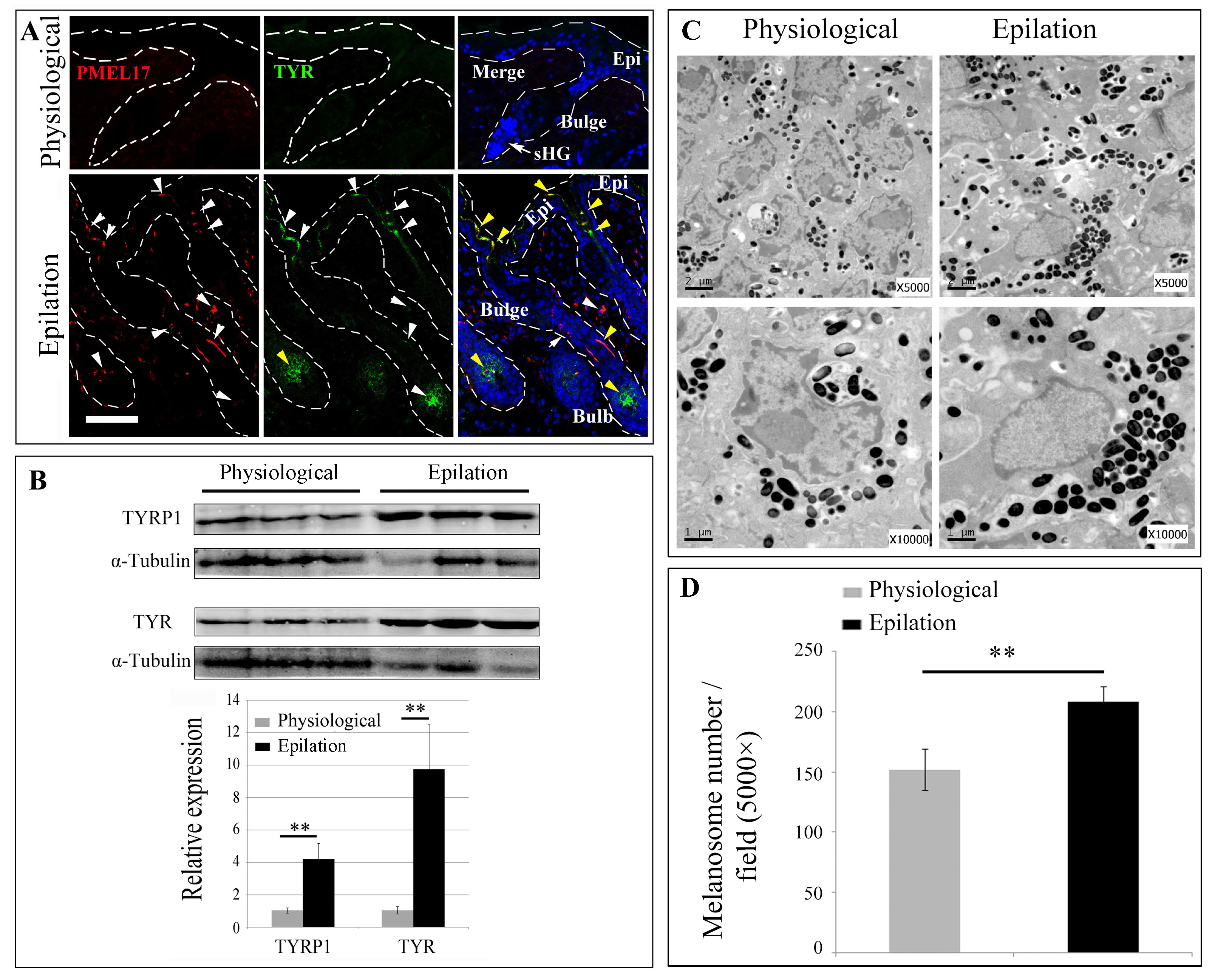


**Figure S4. Epilation induces expression of melanogenesis-related proteins and increases melanosome numbers in hair bulbs.** (A) Immunostaining of anti-PMEL17 and TYR in scalp hair follicles of C57BL/6 mice 5 days after epilation. (B) Western blotting analysis for protein expression of TYRP1 and TYR in the scalp 5 days after epilation. Full-length blots are shown in Figure S9. (C) Melanosomes revealed by transmission electron microscopy (TEM) in the back hair bulbs of mice 7 days after epilation at P21 under the indicated conditions. (D) The bar graphs show the quantification of melanosomes in the back hair bulbs under the indicated conditions.** indicates p<0.01.


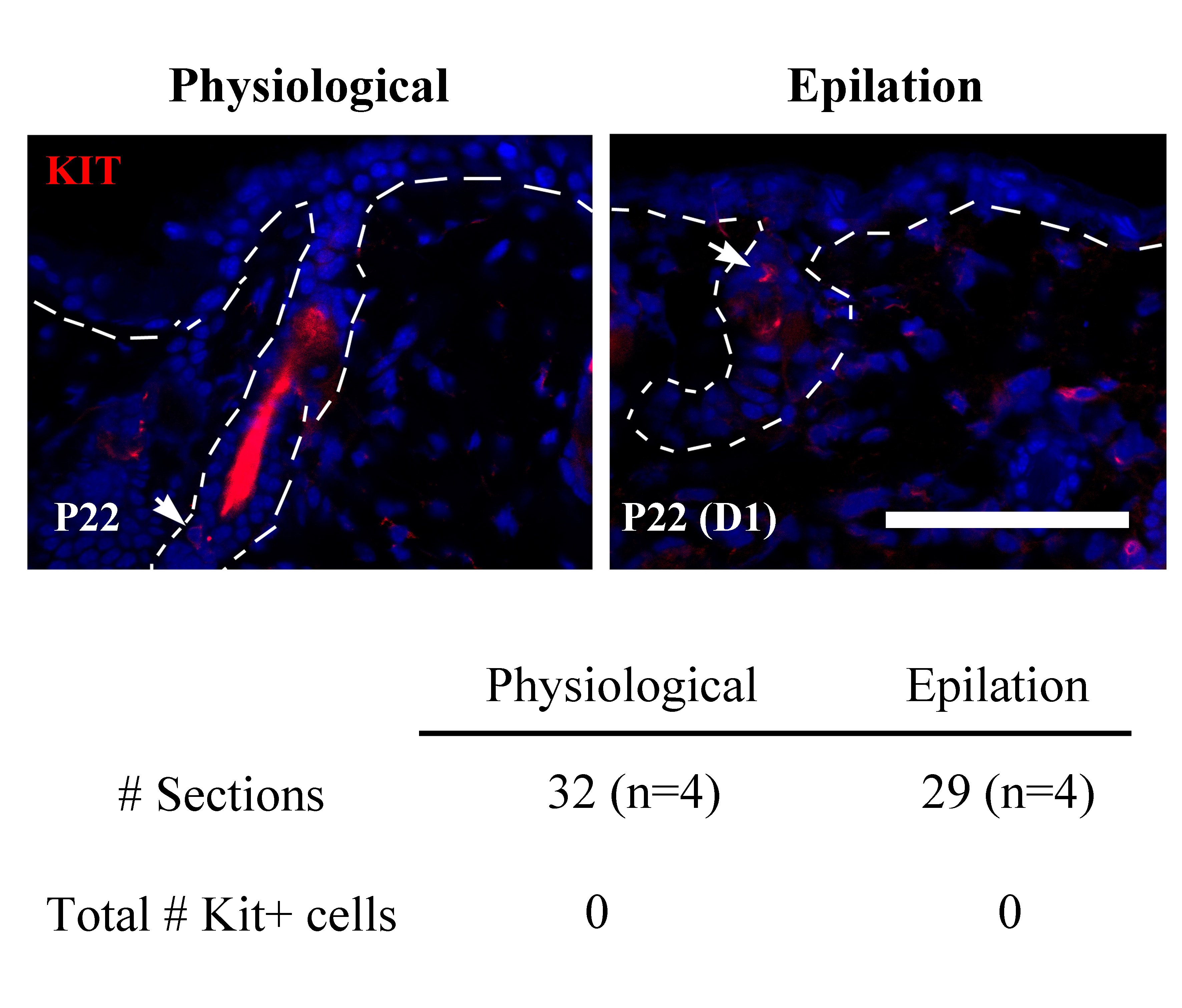
**Figure S5. KIT+ cells are undetectable in the epidermis at P22.** Immunostaining images of anti-KIT in the back skin of mice 1 day after epilation at P21 under the indicated conditions (upper panels). Arrows indicate KIT+ cells in the hair bulge. Quantification shows the number of KIT+ cells in the epidermis (lower panel). Scar bar, 50 μm.


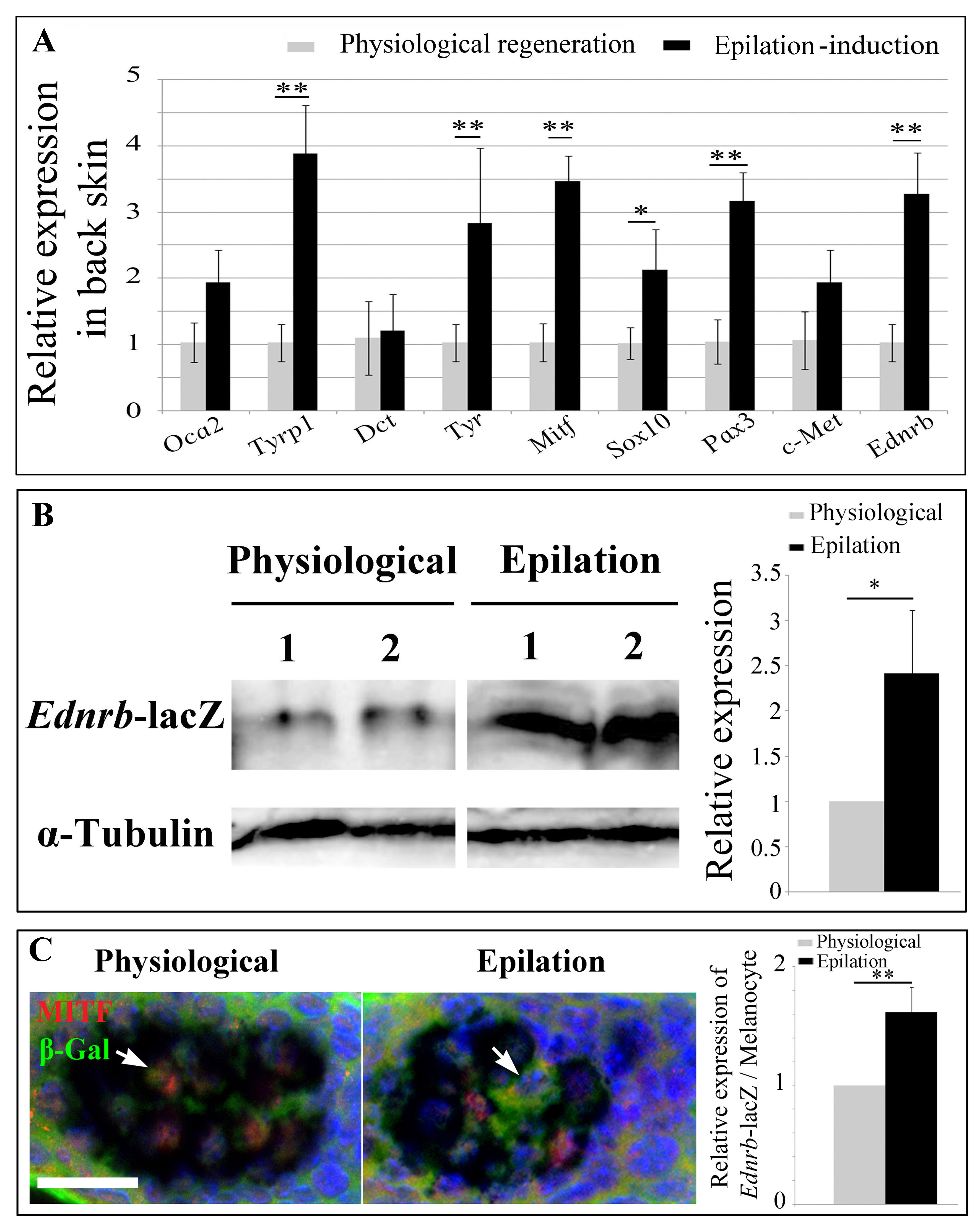


**Figure S6. Epilation induces expression of melanogenesis-related genes.** (A) qRT-PCR analysis shows the expression change of melanogenesis-related genes in the back skin 7 days after epilation. (B) Western blotting analysis for protein expression of *Ednrb*-lacZ in the back skin from *Ednrb^lacZ/+^* mice 7 days after epilation. Full-length gels are shown in Figure S13. (C) Immunostaining of anti-β gal and MITF in the back skin of mice 7 days after epilation at P21 under the indicated conditions. The bar graph (right panels) shows the quantification of anti-β gal signal in melanocytes. (B, C) Paired *t*-test was used to determine the significance of the differences between the population means. Scale bar, 20 μm; * or ** indicates *p*<0.05 or *p*<0.01.


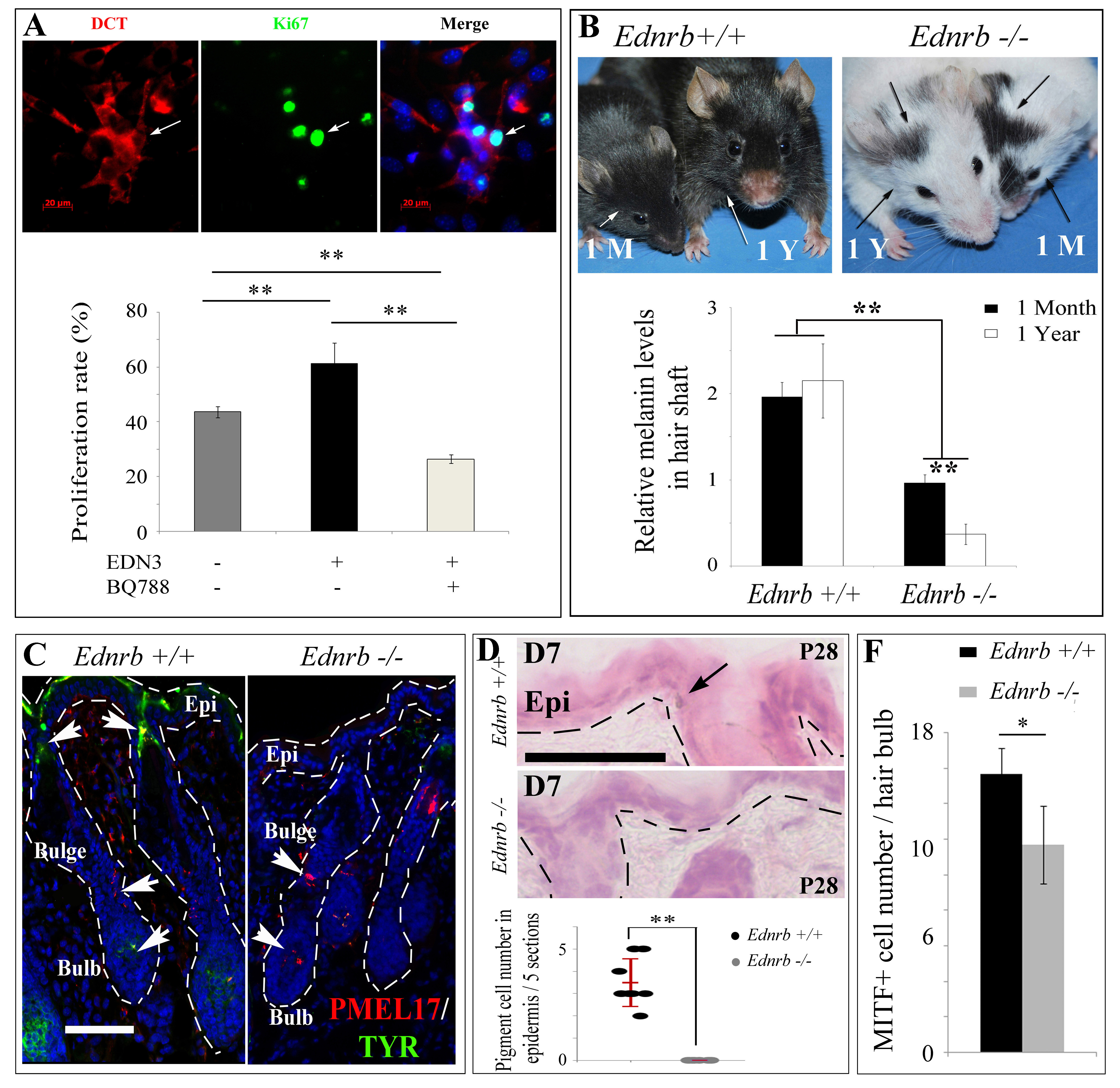


**Figure S7. EDN3/EDNRB is required for McSC proliferation and epilation-induced epidermal melanocyte regeneration.** (A) Anti-DCT and Ki67 immunostaining of melanocyte lineage cells isolated from E16.5 wildtype epidermis (upper panels). The bar graphs (lower panel) show the proliferation rate of melanocyte lineage cells under the indicated conditions. (B) Scalp hair pigmentation of wildtype and *Ednrb-/-* mice at one month and one year after birth (upper panels) and corresponding melanin content of pigmented hair shafts (lower panel). (C) Immunostaining of scalp for anti-PMEL17 and TYR from wildtype and *Ednrb-/-* mice 5 days after epilation. (D) H&E staining of scalp from wildtype and *Ednrb-/-* mice epilated at P21 7 days after epilation. Note lack of pigmented melanocytes in the *Ednrb-/-* epidermis (lower panel). (E) Quantification of MITF-positive cells in scalp hair follicles of wildtype and *Ednrb-/-* mice 7 days after epilation. Epi, epidermis. Scar bar, 50 μm. * indicates p<0.05.


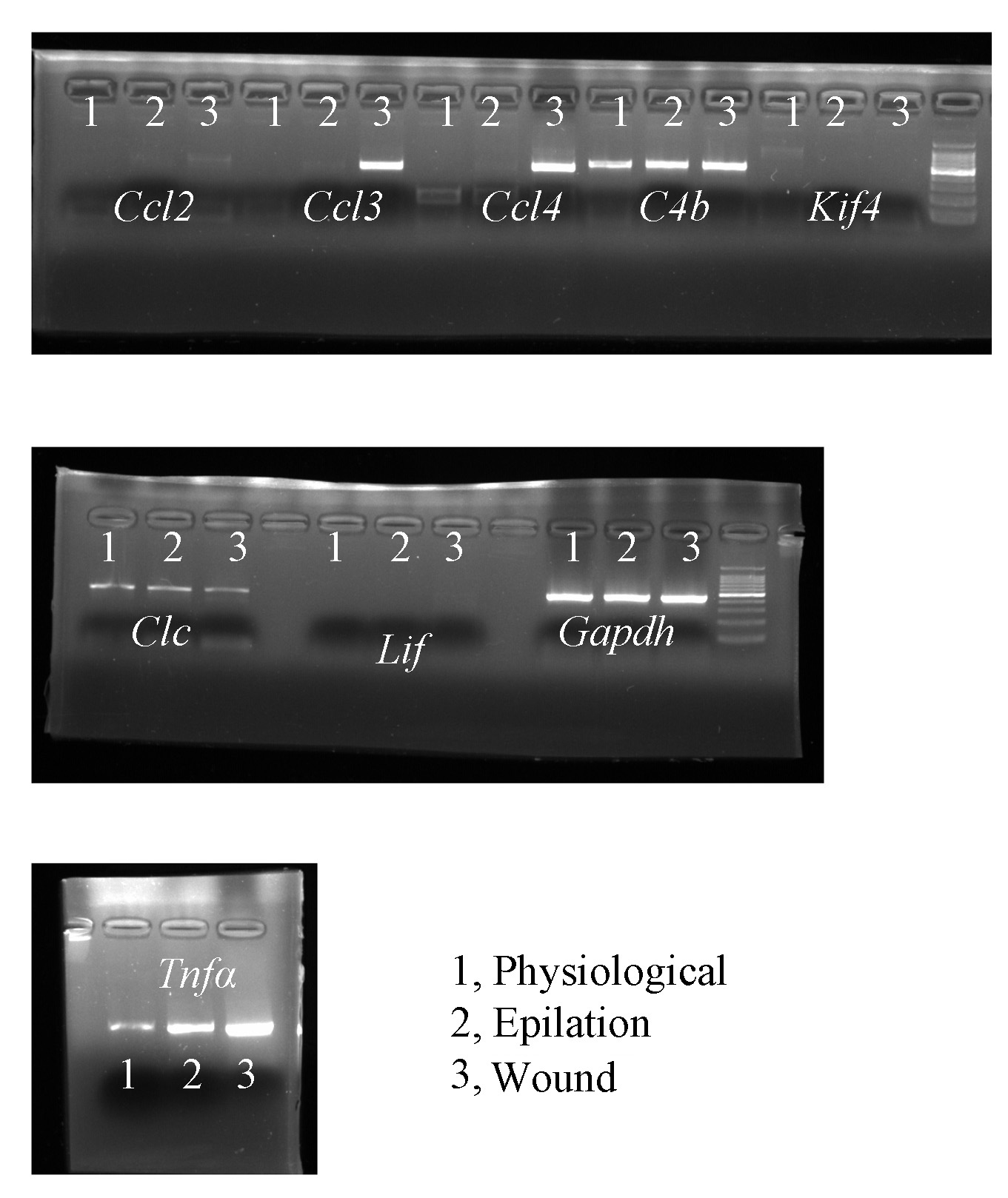


**Figure S8**. The full-length gels for Figure S1A.


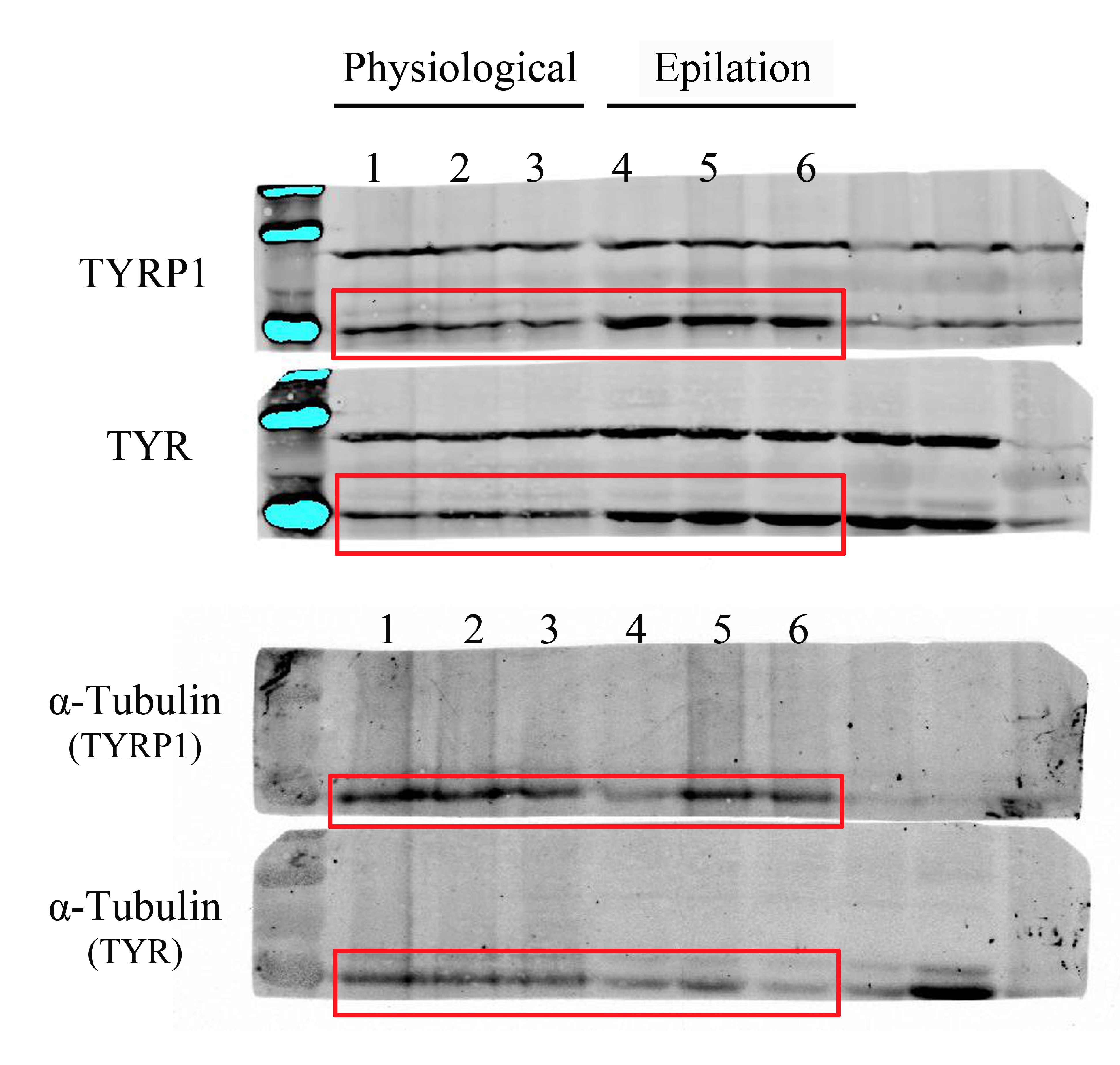


**Figure S9**. The full-length blots for Figure S2B. The red boxes are the cropped blots in the Figure S2B.


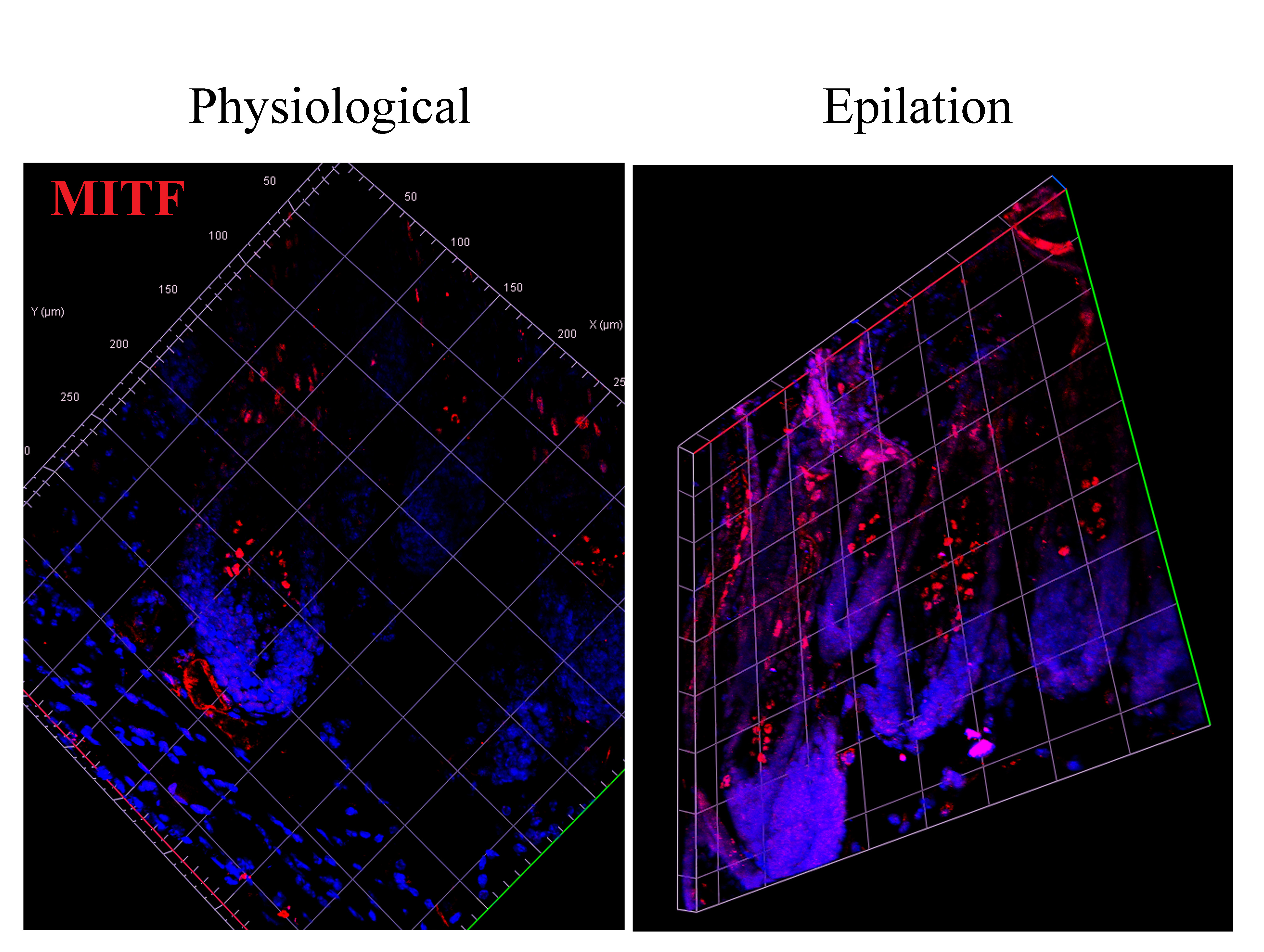


**Figure S10**. The 3D images of MITF-positive cells for Figure 2B.


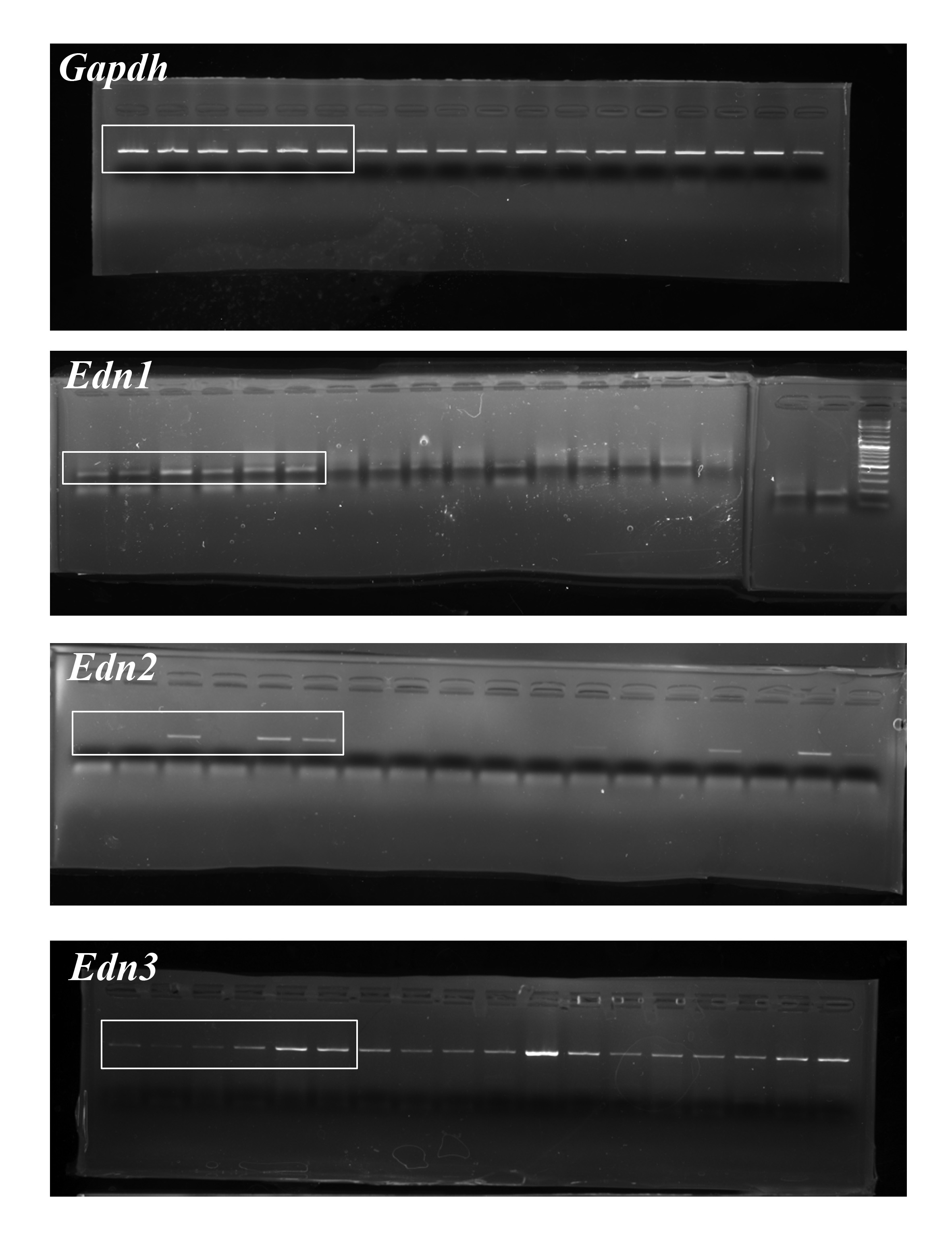


**Figure S11**. The full-length gels for Figure 3A. The white boxes are the cropped gels in Figure 3A.


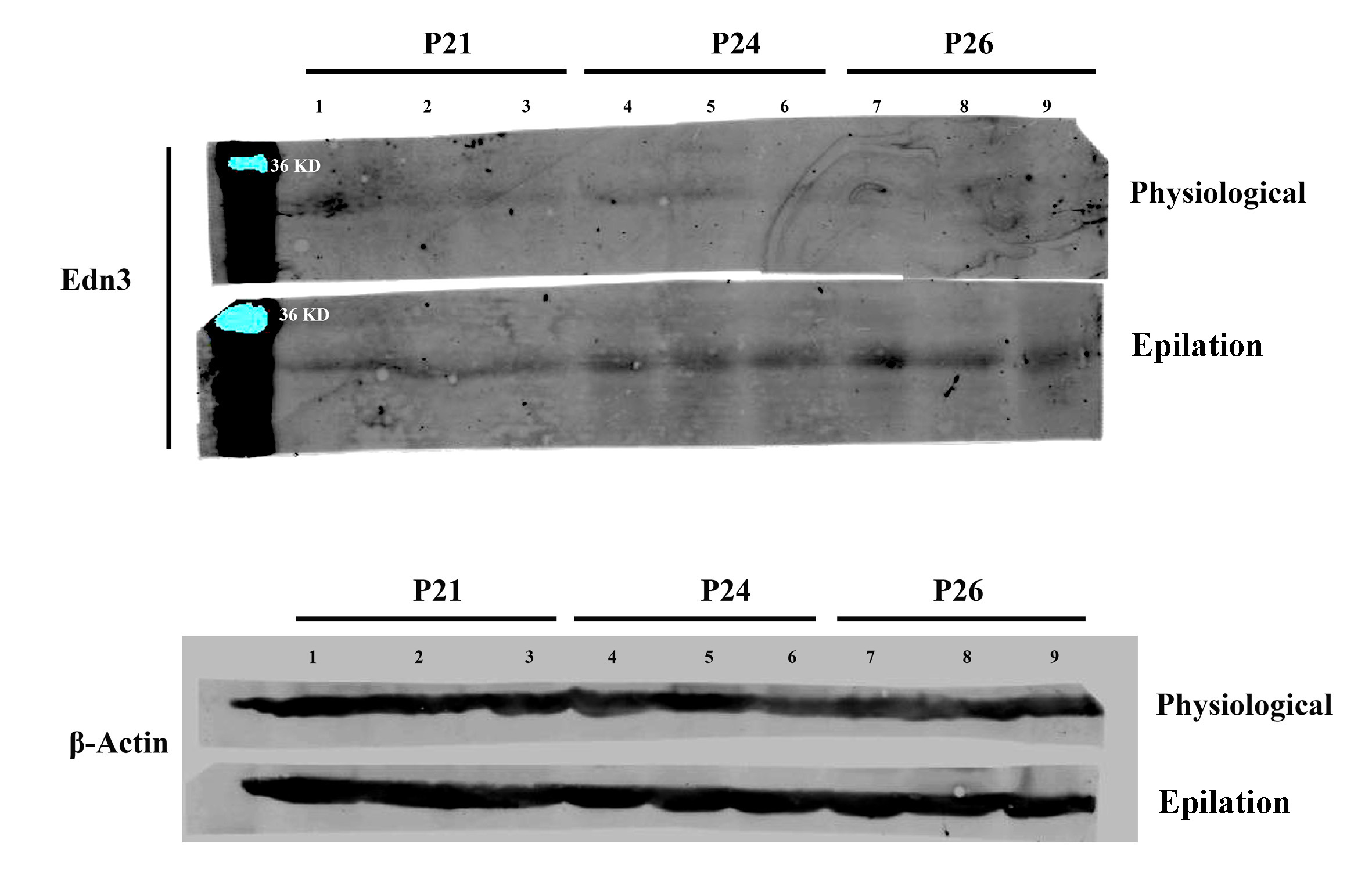


**Figure S12**. The full-length blots for Figure 3B.


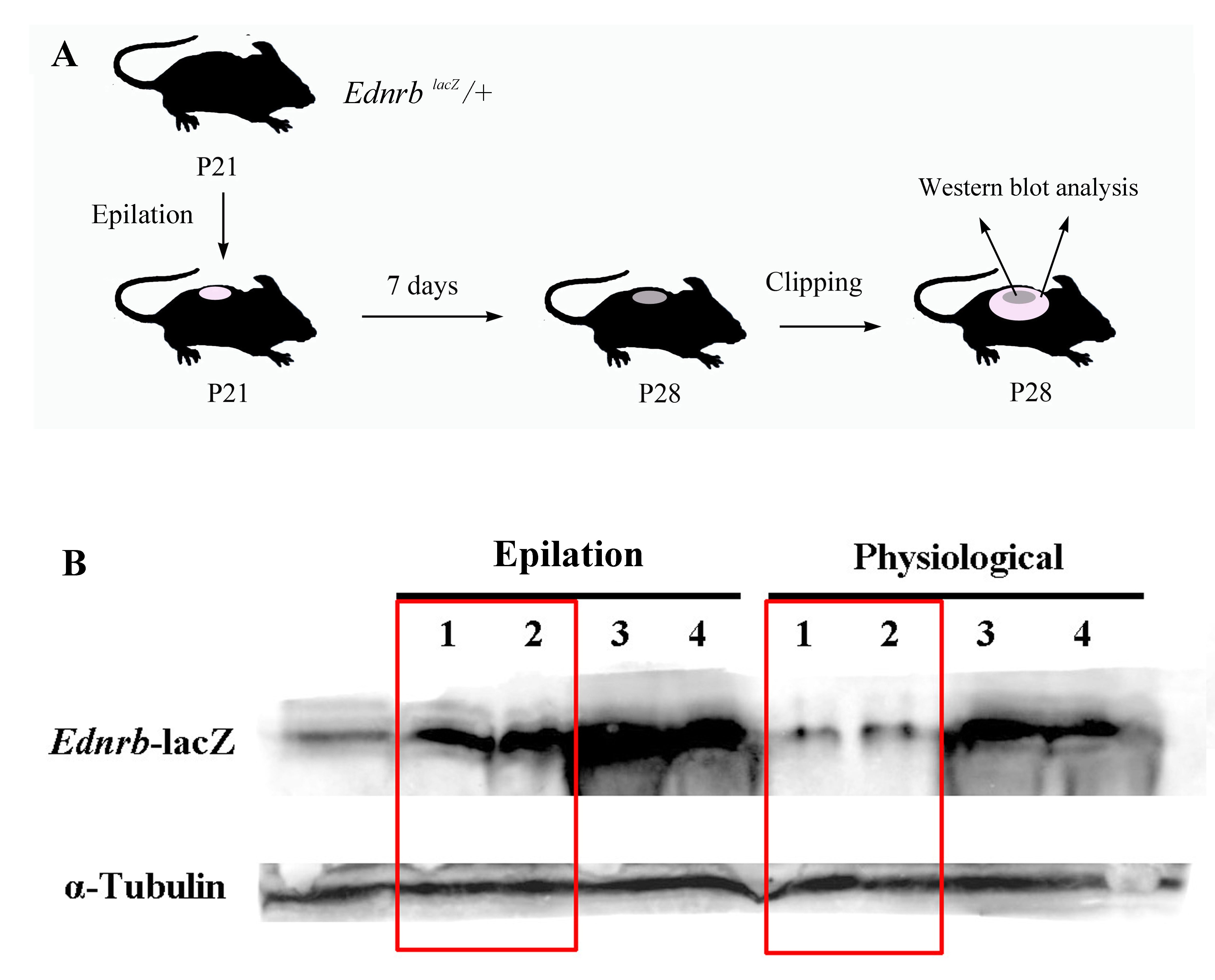


**Figure S13**. (A) The cartoon indicates the experimental epilation procedure in the back. (B) The full-length blots for Figure S6B. The lines marked by the same number represent samples from the same mice under the indicated conditions.
